# Exploring the association between dexmedetomidine and all-cause mortality in mechanically ventilated patients with sepsis through propensity score matching analysis and machine learning algorithms: a MIMIC-IV retrospective study

**DOI:** 10.3389/fcimb.2025.1653883

**Published:** 2026-01-26

**Authors:** Yanxia Wei, Minghui Li, Peng Wang, Jie Zhou, Kejian Lu, Huageng Huang, Yanjuan Huang, Fei Lin

**Affiliations:** 1 Department of Anesthesiology, Guangxi Medical University Cancer Hospital, Nanning, China; 2 Department of Anesthesiology, The Third Affiliated Hospital of Guangxi Medical University, Nanning, China; 3 Department of Anesthesiology, The Second Affiliated Hospital of Guangxi Medical University, Nanning, China

**Keywords:** dexmedetomidine, sepsis, mechanical ventilation, mortality, propensity score matching, machine learning

## Abstract

**Background:**

Sepsis carries high ICU mortality globally, often requiring sedated mechanical ventilation. While some studies suggest dexmedetomidine improves survival in these patients, others contradict this finding. This study evaluates dexmedetomidine’s survival benefit and sedation value for ventilated sepsis cases.

**Methods:**

This retrospective cohort study utilized the MIMIC-IV database and eICU-CRD to analyze mechanically ventilated septic patients. Propensity score matching was employed to balance covariates. Machine learning algorithms were applied to validate dexmedetomidine’s role in predicting mortality.

**Results:**

A propensity score matching analysis was performed for 5176 pairs of patients. The use of dexmedetomidine was associated with a reduced risk of 28-day mortality (13.39% vs. 19.84%, HR: 0.595, *P* < 0.001) and of 180-day all-cause mortality (17.45% vs. 23.18%, HR: 0.632, *P* < 0.001). However, dexmedetomidine use was also associated with longer hospital (median 15.08 days vs. 10.2 days, *P* < 0.001) and ICU stays (median 6.81 days vs. 4.0 days, *P* < 0.001). Moreover, the duration of mechanical ventilation was significantly longer in the dexmedetomidine group (median 78 h vs. 51.00 h, *P* < 0.001). Dexmedetomidine was included among the significant features identified with the Boruta algorithm, and of the five machine learning models built using the 20 most important features (including dexmedetomidine), the model constructed on the basis of the Random Forest algorithm performed the best (training set: AUC = 0.781; test set: AUC = 0.811; eICU-CRD set: AUC = 0.820). SHapley Additive exPlanations (SHAP) revealed that comorbid acute kidney injury (AKI) was the most important predictor of mortality among mechanically ventilated septic patients. This was followed by the use of opioids, PaO_2_, and the SOFA score, with the use of dexmedetomidine relatively closely behind.

**Conclusions:**

Dexmedetomidine use significantly reduces short-term mortality in mechanically ventilated patients with sepsis but prolongs the hospital and ICU length of stay (LOS) and duration of mechanical ventilation. Administering dexmedetomidine within 48 hours and maintaining an infusion rate at or below 0.6 μg/kg/h appears to be more beneficial. Moreover, dexmedetomidine use strongly influences mortality in these patients.

## 1 Introduction

Sepsis is a life-threatening form of organ dysfunction triggered by a dysregulated host response to infection, and among intensive care unit (ICU) patients, its morbidity and mortality rates are consistently high worldwide. According to data from the Global Burden of Disease Study, 11 million sepsis-related deaths occurred worldwide in 2017, accounting for 19.7% of all deaths (Rudd et al., 2020). Although mechanical ventilation is effective in improving oxygenation and reducing the risk of lung injury as core support methods for patients with sepsis combined with respiratory failure, its use is often accompanied by the need for sedation. The Society of Critical Care Medicine (SCCM) guidelines recommend propofol or dexmedetomidine as the preferred sedative for mechanically ventilating patients (Devlin et al., 2018), but the difference in efficacy between the two remains controversial. Dexmedetomidine, a highly selective α_2_-adrenoceptor agonist, has sedative and anxiolytic properties, can preserve spontaneous respiration, and has the potential to reduce the inflammatory response in sepsis and protect organ function through multiple mechanisms, including inhibition of the NF-κB signaling pathway, modulation of macrophage polarization and improvement in microcirculation (Zhang et al., 2018; Liu et al., 2019; Mei et al., 2021). However, the differences among clinical findings regarding this drug has led to different evaluations of its efficacy: some systematic meta-analyses have shown that dexmedetomidine reduces 28-day mortality and shortens the duration of mechanical ventilation (Chen et al., 2020; Wang et al., 2021), whereas a recent multicenter clinical randomized controlled trial (RCT) (Kawazoe et al., 2017) reported no significant survival benefit with the use of dexmedetomidine over propofol. This contradiction may stem from differences in the designs of the studies, including the identification of confounding factors such as the baseline characteristics of the population, choice of sedation regimen, and mechanical ventilation parameter settings.

The limitations of the available clinical evidence further emphasize the need for this study. First, the methodological heterogeneity among the studies included in most systematic meta-analyses, such as the failure of some trials to differentiate between mechanically ventilated and nonmechanically ventilated patients or the inclusion of benzodiazepines in the control group (Kawazoe et al., 2017; Zhang et al., 2019), limit extrapolation of the results. Second, the pathological heterogeneity of the sepsis itself (e.g., source of infection, degree of organ dysfunction) was not adequately stratified and may have masked the subgroup effects of the use of dexmedetomidine.

In recent years, artificial intelligence (AI) technology has gradually become an important tool in the fields of disease prediction and outcome assessment. Compared with traditional disease severity scoring systems [e.g., the Sequential Organ Failure Assessment (SOFA) score and Acute Physiology and Chronic Health Evaluation II (APACHE II)], machine learning-based models are able to more accurately identify high-risk patients and predict clinical outcomes by deeply mining nonlinear associations and interaction effects in the data (Burki, 2021; Bi et al., 2022; Li et al., 2022). For example, eXtreme gradient boosting (XgBoost)-based models provide the highest mortality prediction performance for acute kidney injury (AKI) patients requiring renal replacement therapy, significantly outperforming the predictive efficacy of traditional scoring systems (Chang et al., 2022). Therefore, it is necessary to use interpretable machine learning methods to verify whether dexmedetomidine has an important influence on mortality in mechanically ventilated patients with sepsis.

The goal of this study was to evaluate the effects of dexmedetomidine on short-term mortality and outcomes on the basis of real-world data from mechanically ventilated patients with sepsis in the Medical Information Intensive Care Database IV (MIMIC-IV, version 3.1) and eICU Collaborative Research Database (eICU-CRD, version 2.0). By integrating multidimensional clinical variables with advanced statistical methods, we explored the factors influencing the outcomes of mechanically ventilated patients with sepsis to provide an evidence basis for optimizing sedation strategies and promoting precision medicine.

## 2 Materials and methods

### 2.1 Study design and database access

A retrospective cohort study was performed to investigate whether dexmedetomidine improves survival in mechanically ventilated patients with sepsis and to explore the importance of dexmedetomidine in the sedation of these patients. Patient data were obtained from MIMIC-IV (version 3.1) and eICU-CRD (version 2.0). MIMIC-IV is a database that contains comprehensive medical record information on patients admitted to the ICU of Beth Israel Deaconess Medical Center in Boston between 2008 and 2022, whereas the eICU-CRD incorporates data from multiple U.S. institutions, representing a broader range of patient populations and clinical practices. The collection of patient information and the creation of the database were reviewed by the Beth Israel Deaconess Medical Center Institutional Review Board (IRB), which waived the need for written informed consent and approved the data-sharing plan; no additional ethical approval was required for this study (Johnson et al., 2016). Author Wei YX is certified and licensed by the Collaborative Institutional Training Initiative (CITI) to use the MIMIC-IV database and eICU-CRD (certification number: 67968198) in accordance with the relevant regulations. This study was conducted in accordance with the principles of the Declaration of Helsinki.

### 2.2 Patient selection

Patients who were diagnosed with sepsis within 24 h of admission to the ICU were included in this study. According to the sepsis 3.0 definition, sepsis is defined as life-threatening organ dysfunction due to an imbalance in response to infection, requiring a confirmed or suspected infection and a sudden increase in the total SOFA score of 2 or more points (Singer et al., 2016). The exclusion criteria were as follows: (1) age < 18 years; (2) nonfirst hospital and ICU admission; (3) ICU length of stay (LOS) < 24 h; (4) nonmechanical ventilation; (5) no use of the four sedatives (dexmedetomidine, midazolam, propofol, etomidate) during the ICU stay; and (6) abnormal recording of vital signs.

Eligible patients were stratified into two cohorts: (I) dexmedetomidine-exposed patients (DEX group) and (II) nondexmedetomidine-exposed patients, that is, those receiving propofol, midazolam, or etomidate (Non-DEX group). Dexmedetomidine was permitted as adjunctive therapy in the DEX group, whereas dexmedetomidine was explicitly not permitted in the non-DEX group.

### 2.3 Data extraction

The following variables were extracted from the MIMIC-IV database (version 3.1) and eICU-CRD (version 2.0): demographic characteristics, including sex, age, ethnicity; disease severity, as reported by the SOFA score within 24 h of ICU admission; laboratory parameters (first measurement in the ICU), including white blood cell (WBC) count, red blood cell (RBC) count, hematocrit, hemoglobin level, platelet count, red cell distribution width (RDW), creatinine level, blood urea nitrogen (BUN) level, international normalized ratio (INR), prothrombin time (PT), partial thromboplastin time (PTT), partial pressure of arterial carbon dioxide (PaCO_2_), and partial pressure of arterial oxygen (PaO_2_); vital signs (first measurement in the ICU), including blood pressure, heart rate, respiratory rate (RR), saturation of peripheral oxygen (SpO_2_); comorbidities, including hypertension, cirrhosis, pneumonia, cerebrovascular accident (CVA), cancer, diabetes, heart failure, myocardial infarction (MI), ischemic heart disease (IHD), and chronic obstructive pulmonary disease (COPD), Acute kidney injury (AKI; AKI was defined according to the KDIGO staging criteria); and medication use, including the use of antibiotics (cephalosporins, aminoglycosides, macrolides, etc.), vasopressors (norepinephrine, epinephrine, epinephrine, dopamine, etc.), glucocorticoids (methylprednisolone, dexamethasone, hydrocortisone, etc.), opioids (fentanyl, morphine), and sedatives (dexmedetomidine, propofol, midazolam, etomidate), duration of administration and cumulative dose of dexmedetomidine. Additional outcomes included mechanical ventilation duration; ICU and hospital LOS; and survival; ventilator-free days at 28 days (defined as days alive and free from mechanical ventilation within the first 28 days) and ICU-free days at 28 days (defined as days alive and out of the ICU within the first 28 days with deaths coded as 0).

### 2.4 Outcomes

The primary outcome was 28-day all-cause mortality. The secondary outcome was 180-day all-cause mortality. Exploratory outcomes included hospital LOS, ICU LOS, mechanical ventilation duration (hours), dexmedetomidine duration (hours), infusion rate of dexmedetomidine (μg/kg/h) and the importance value of dexmedetomidine in machine learning models for predicting 28-day mortality in mechanically ventilated patients with sepsis.

### 2.5 Statistical analysis

This was a retrospective cohort study. After screening, there were no missing values for any categorical variables in this study, continuous variables missing more than 20% of their values were excluded and are shown in **Supplementary Table S1**, and the null values for continuous variables missing fewer than 20% of their values were interpolated using the K-nearest neighbor (KNN) method. In this study, we used propensity score matching (PSM: caliper 0.05) to reduce differences in baseline characteristics between the two groups. We calculated the standardized mean difference (SMD) to assess the reported effectiveness of PSM in mitigating these differences. Patients were divided into two groups on the basis of whether dexmedetomidine was used. Normally distributed continuous variables were compared between the groups with the t test and are expressed as the means ± standard deviations. Nonnormally distributed continuous variables were compared between the groups with the rank-sum test and are expressed as medians (Q1, Q3). Categorical variables were compared with analysis of variance and are expressed as n (%).

A Cox regression model was used to analyze the association between dexmedetomidine administration and 28- and 180-day all-cause mortality. Hazard ratios (HRs) and 95% confidence intervals (CIs) were calculated for these associations using Cox regression modelling. Least absolute shrinkage and selection operator (LASSO) regression was used to address the multiple covariances among the variables. First, LASSO regression was performed out to select the variables without covariates (coeff_lamda≠0), after which Cox regression modelling was performed with the retained variables. Three Cox models were generated: Model 1: uncorrected; Model 2: corrected for age, ethnicity, and SOFA score; and Model 3: corrected for nonlinearly related covariates. Kaplan–Meier analysis was performed to generate survival curves for 28- and 180-day mortality in mechanically ventilated patients with sepsis.

Additionally, HRs with 95% CIs were calculated for each subgroup by duration of dexmedetomidine administration and infusion rate.

To further evaluate the robustness of the model and the reliability of our conclusions, we conducted multiple sensitivity analyses. First, Cox regression models were applied in a subgroup of mechanically ventilated patients with sepsis and SOFA scores greater than 8. Second, a fully adjusted model was fitted after excluding patients with missing continuous variables (**Supplementary Table S1**). Third, patients were stratified into four medication−based groups (non−dexmedetomidine plus non−propofol, dexmedetomidine plus propofol, dexmedomidine without propofol, and other combinations) and analyzed using Cox proportional−hazards regression. Finally, we performed additional validation stratified by AKI stage.

The study also analyzed the data of mechanically ventilated patients with sepsis grouped according to the following variables: age (≤ 65 and > 65 years), sex, ethnicity, SOFA score (≤ 8 and > 8), the presence or absence of hypertension, the presence or absence of AKI, the presence or absence of cancer, the use of vasopressors, the use of opioids, and the duration of mechanical ventilation (≤ 50 h and > 50 h). All subgroup analyses were conducted after adjusting for the following covariates: antibiotic use, glucocorticoid use, systolic blood pressure (SBP), diastolic blood pressure (DBP), heart rate, RR, SpO_2_, PaO_2_, and PaCO_2_. The HRs and 95% CIs were calculated for each subgroup.

The Boruta algorithm was used to determine the most important features that affected 28-day all-cause mortality in mechanically ventilated patients with sepsis; the 20 most important features identified with the algorithm were included in the construction and validation of five machine learning models [based on the Random Forest, Conditional Inference Trees (Ctree), Gradient Boosting Machines (GBM), Generalized Additive Model Boosting (gamBoost), and eXtreme Gradient Boosting (Xgboost)] for predicting the 28-day mortality rate among mechanically ventilated patients with sepsis. The MIMIC-IV dataset was divided into training and validation sets at a ratio of 7:3. The performance of the models was assessed with receiver operating characteristic (ROC) curve analysis, including calculation of the area under the curve (AUC), specificity, and sensitivity. Decision curve analysis (DCA) was used to assess the clinical value of the predictive models, and the calibration curve was used to assess the agreement between predicted probabilities and observed outcomes. The eICU-CRD cohort served as an external validation set for the Random Forest model. SHapley Additive exPlanations (SHAP) was applied to further interpret the predictive performance of the model.

DecisionLinnc (version 1.1.5.6), a platform that integrates R and Python programming language environments, was used to conduct visual data processing and data analysis (DecisionLinnc Core Team, 2024). *P* < 0.05 was considered to indicate statistical significance.

## 3 Results

### 3.1 Baseline patient characteristics

**Figure 1:**
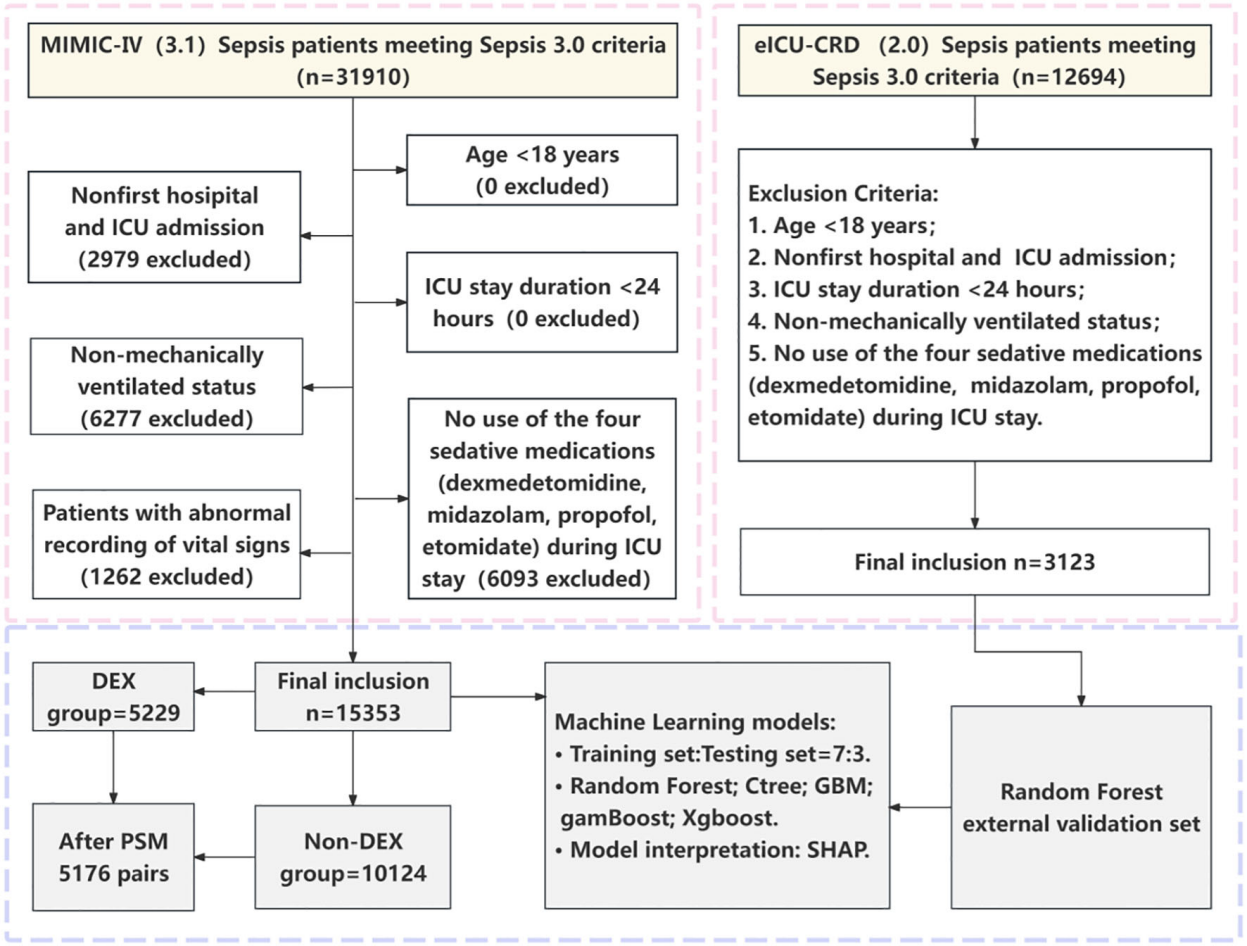
Flowchart of this study. DEX, dexmedetomidine; PSM, propensity score matching; Ctree, Conditional Inference Trees; GBM, Gradient Boosting Machines; gamBoost, Generalized Additive Model Boosting; Xgboost, eXtreme Gradient Boosting; SHAP, SHapley Additive exPlanations.

**Figure 2:**
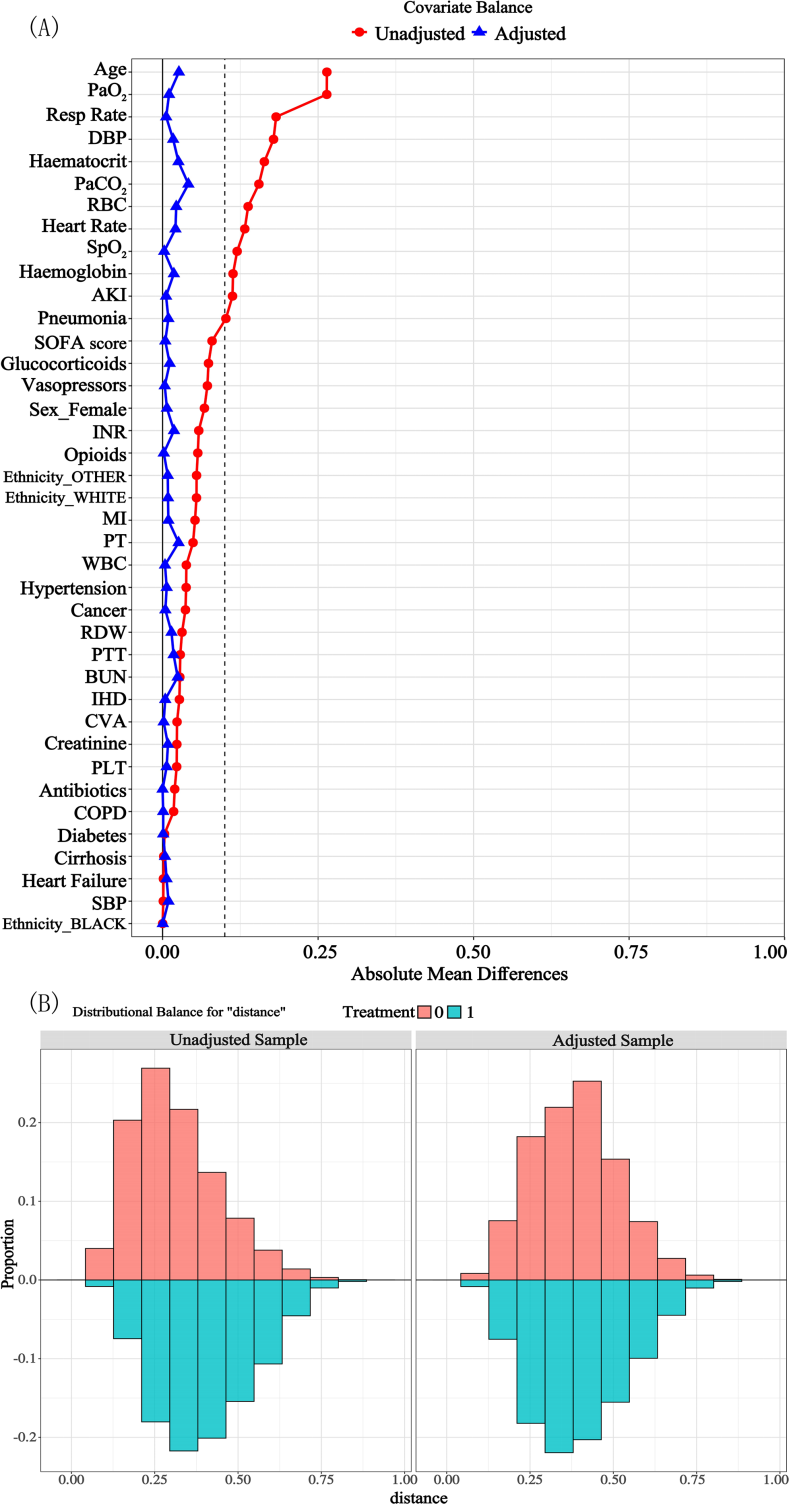
Comparison chart of the distribution balance of variables before and after PSM. **(A)** PSM adjustment for covariates absolute average difference. This graph shows the horizontal axis represents the absolute mean difference value, while the vertical axis indicates the specific covariate names. The dotted line is used to assist in determining the critical threshold range for the differences; **(B)** this graph shows the distribution ratios of different treatment groups for the variable in the unadjusted sample and the adjusted sample. The horizontal axis represents the values of distance, and the vertical axis represents the proportion. PSM, propensity score matching; SMD, standardized mean difference; SOFA, Sequential Organ Failure Assessment; WBC, white blood cell; RBC, red blood cell; RDW, red blood cell distribution width; BUN, blood urea nitrogen; INR, international normalized ratio; PT, prothrombin time; PTT, partial thromboplastin time; PaCO_2_, partial pressure of carbon dioxide in arterial blood; PaO_2_, partial pressure of oxygen in arterial blood; SBP, systolic blood pressure; DBP, diastolic blood pressure; Resp rate, respiratory rate; SpO_2_, saturation of peripheral oxygen; AKI, acute kidney injury; CVA, cerebrovascular accident; MI, myocardial infarction; IHD, ischemic heart disease; COPD, chronic obstructive pulmonary disease.

The data of 31910 patients with sepsis during the study period were extracted (**Figure 1**); of these patients, 15353 were eligible for analysis following application of the exclusion criteria. Of these, 5229 patients were allocated to the DEX group, and 10124 were allocated to the Non-DEX group. The median age of the patients in the two groups after PSM was 64 years, and most patients in both groups were male (DEX group: 66.09%, Non-DEX group: 65.07%) and of white ethnicity (DEX group: 60.49%, Non-DEX group: 61.65%). Before PSM, there were significant differences between the DEX and Non-DEX groups in terms of sex, age, ethnicity, SOFA score, laboratory tests, vital signs, comorbidities, and use of therapeutic medications (*P* < 0.05), while no statistically significant differences were identified in terms of SBP, comorbid cirrhosis, diabetes mellitus or heart failure (*P* > 0.05). After PSM, 5176 patients who received dexmedetomidine were matched with 5176 patients who did not, and the baseline characteristics of the matched individuals were evenly distributed (**Table 1**). PSM successfully balanced baseline characteristics between the groups, with every standardized mean difference (SMD) falling below the 0.1 threshold (**Table 1**; **Figure 2**).

**Table 1:** Baseline characteristics of the two groups before and after PSM.

Characteristics	Before PSM				After PSM			
	DEX group (n = 5229)	Non-DEX group (n = 10124)	SMD	*P*	DEX group (n = 5176)	Non-DEX group (n = 5176)	SMD	*P*
Sex [Male, n (%)]	3460.00 (66.17)	6018.00 (59.44)	0.140	< 0.001	3421 (66.09)	3368 (65.07)	0.02	0.273
Age (years)	64.00 (52.00-74.00)	68.00 (58.00-78.00)	0.27	< 0.001	64.00 (53.00-74.00)	64.00 (53.00-74.00)	0.02	0.187
Ethnicity, n(%)			0.12	< 0.001			0.03	0.437
White	3159.00 (60.41)	6668.00 (65.86)			3131 (60.49)	3191 (61.65)		
Black	361.00 (6.90)	700.00 (6.91)			360 (6.96)	360 (6.96)		
Other	1709.00 (32.68)	2756.00 (27.22)			1685 (32.55)	1625 (31.39)		
SOFA score	6.00 (4.00-9.00)	6.00 (4.00-8.00)	-0.08	< 0.001	6.00 (4.00-9.00)	6.00 (4.00-9.00)	0.00	0.420
Laboratory tests								
WBC count (K/µL)	12.20 (8.80-16.70)	12.00 (8.50-16.30)	-0.04	0.001	12.20 (8.80-16.70)	12.20 (8.70-16.70)	-0.01	0.596
RBC count (m/µL)	3.53 (2.98-4.14)	3.40 (2.91-3.95)	-0.14	< 0.001	3.53 (2.98-4.14)	3.52 (2.99-4.07)	-0.02	0.273
Hematocrit (%)	32.20 (27.40-37.50)	30.90 (26.60-35.70)	-0.17	< 0.001	32.10 (27.30-37.50)	32.00 (27.40-36.80)	-0.02	0.237
Hemoglobin (g/dL)	10.60 (8.90-12.40)	10.20 (8.80-11.90)	-0.12	< 0.001	10.60 (8.90-12.40)	10.50 (9.00-12.20)	-0.01	0.433
Platelet count (K/µL)	178.00 (129.00-241.00)	172.00 (123.00-237.00)	-0.02	0.002	178.00 (128.00-240.70)	178.00 (127.00-243.00)	0.01	0.959
RDW (%)	14.20 (13.30-15.70)	14.30 (13.40-15.80)	0.03	< 0.001	14.20 (13.30-15.70)	14.30 (13.30-15.70)	0.00	0.050
Creatinine (mg/dL)	1.00 (0.80-1.50)	1.00 (0.70-1.40)	-0.02	< 0.001	1.00 (0.80-1.50)	1.00 (0.70-1.50)	0.00	0.911
BUN (mg/dL)	19.00 (13.00-30.00)	19.00 (14.00-30.00)	0.03	0.015	19.00 (13.00-30.00)	19.00 (13.00-31.00)	0.01	0.105
INR (ratio)	1.30 (1.20-1.50)	1.30 (1.20-1.60)	0.06	< 0.001	1.30 (1.20-1.50)	1.30 (1.20-1.60)	0.01	0.018
PT (s)	14.30 (12.60-16.70)	14.90 (13.20-17.10)	0.05	< 0.001	14.30 (12.70-16.70)	14.60 (13.00-16.95)	0.01	< 0.001
PTT (s)	30.60 (27.10-36.90)	31.40 (27.60-38.20)	0.03	< 0.001	30.60 (27.10-36.80)	31.20 (27.40-37.70)	0.01	< 0.001
PaCO_2_ (mmHg)	42.00 (37.00-49.00)	41.00 (36.00-46.00)	-0.16	< 0.001	42.00 (37.00-48.45)	41.00 (36.00-48.00)	-0.04	< 0.001
PaO_2_ (mmHg)	119.00 (64.00-254.00)	170.00 (84.65-309.00)	0.26	< 0.001	120.00 (64.00-256.00)	128.00 (71.00-252.00)	0.02	0.009
Vital signs								
SBP (mmHg)	116.00 (101.00-131.00)	115.00 (101.00-132.00)	0.00	0.804	116.00 (101.00-131.00)	115.00 (101.00-132.00)	-0.01	0.463
DBP (mmHg)	66.00 (56.00-77.00)	62.00 (53.00-74.00)	-0.18	< 0.001	66.00 (56.00-77.00)	65.00 (55.00-77.00)	-0.01	0.297
Heart rate (bpm)	88.00 (77.00-102.00)	84.00 (75.00-98.00)	-0.13	< 0.001	88.00 (77.00-102.00)	87.00 (76.00-102.00)	-0.03	0.086
Resp rate (bpm)	18.00 (15.00-23.00)	17.00 (14.00-21.00)	-0.19	< 0.001	18.00 (15.00-23.00)	18.00 (15.00-23.00)	-0.01	0.317
SpO_2_ (%)	99.00 (95.00-100.00)	100.00 (96.00-100.00)	0.12	< 0.001	99.00 (96.00-100.00)	99.00 (96.00-100.00)	0.03	< 0.001
Comorbidities, n (%)								
Hypertension	2217.00 (42.40)	4676.00 (46.19)	0.08	< 0.001	2195 (42.41)	2219 (42.87)	0.01	0.633
AKI	2411.00 (46.11)	3530.00 (34.87)	0.23	< 0.001	2371 (45.81)	2304 (44.51)	0.03	0.186
Cirrhosis	483.00 (9.24)	954.00 (9.42)	0.01	0.707	478 (9.23)	487 (9.41)	0.01	0.761
Pneumonia	2262.00 (43.26)	3349.00 (33.08)	0.21	< 0.001	2224 (42.97)	2135 (41.25)	0.03	0.076
CVA	359 (6.87)	930 (9.19)	0.09	< 0.001	358 (6.92)	364 (7.03)	0.00	0.817
Cancer	595 (11.38)	1524 (15.05)	0.11	< 0.001	595 (11.50)	594 (11.48)	0.00	0.975
Diabetes	1475 (28.21)	2887 (28.52)	0.01	0.688	1458 (28.17)	1450 (28.01)	0.00	0.861
Heart Failure	1366 (26.12)	2658 (26.25)	0.00	0.861	1351 (26.10)	1337 (25.83)	0.01	0.754
MI	615 (11.76)	663 (6.55)	0.18	< 0.001	601 (11.61)	544 (10.51)	0.04	0.074
IHD	2012 (38.48)	4169 (41.18)	0.06	0.001	1994 (38.52)	1985 (38.35)	0.00	0.856
COPD	840 (16.06)	1447 (14.29)	0.05	0.003	828 (16.00)	817 (15.78)	0.01	0.767
Interventions, n (%)								
Antibiotics	5089 (97.32)	9656 (95.38)	0.10	< 0.001	5036 (97.30)	5029 (97.16)	0.01	0.675
Vasopressors	4367 (83.52)	7726 (76.31)	0.18	< 0.001	4315 (83.37)	4308 (83.23)	0.00	0.854
Glucocorticoids	1675 (32.03)	2496 (24.65)	0.16	< 0.001	1647 (31.82)	1592 (30.76)	0.02	0.244
Opioids	2569 (49.13)	5547 (54.79)	0.11	< 0.001	2545 (49.17)	2636 (50.93)	0.04	0.074

PSM, propensity score matching; SMD, standardized mean difference; SOFA, Sequential Organ Failure Assessment; WBC, white blood cell; RBC, red blood cell; RDW, red blood cell distribution width; BUN, blood urea nitrogen; INR, international normalized ratio; PT, prothrombin time; PTT, partial thromboplastin time; PaCO_2_, partial pressure of carbon dioxide in arterial blood; PaO_2_, partial pressure of oxygen in arterial blood; SBP, systolic blood pressure; DBP, diastolic blood pressure; Resp rate, respiratory rate; SpO_2_, saturation of peripheral oxygen; AKI, acute kidney injury; CVA, cerebrovascular accident; MI, myocardial infarction; IHD, ischemic heart disease; COPD, chronic obstructive pulmonary disease.

### 3.2 Dexmedetomidine use reduces 28-day and 180-day all-cause mortality in mechanically ventilated patients with sepsis

**Figure 3:**
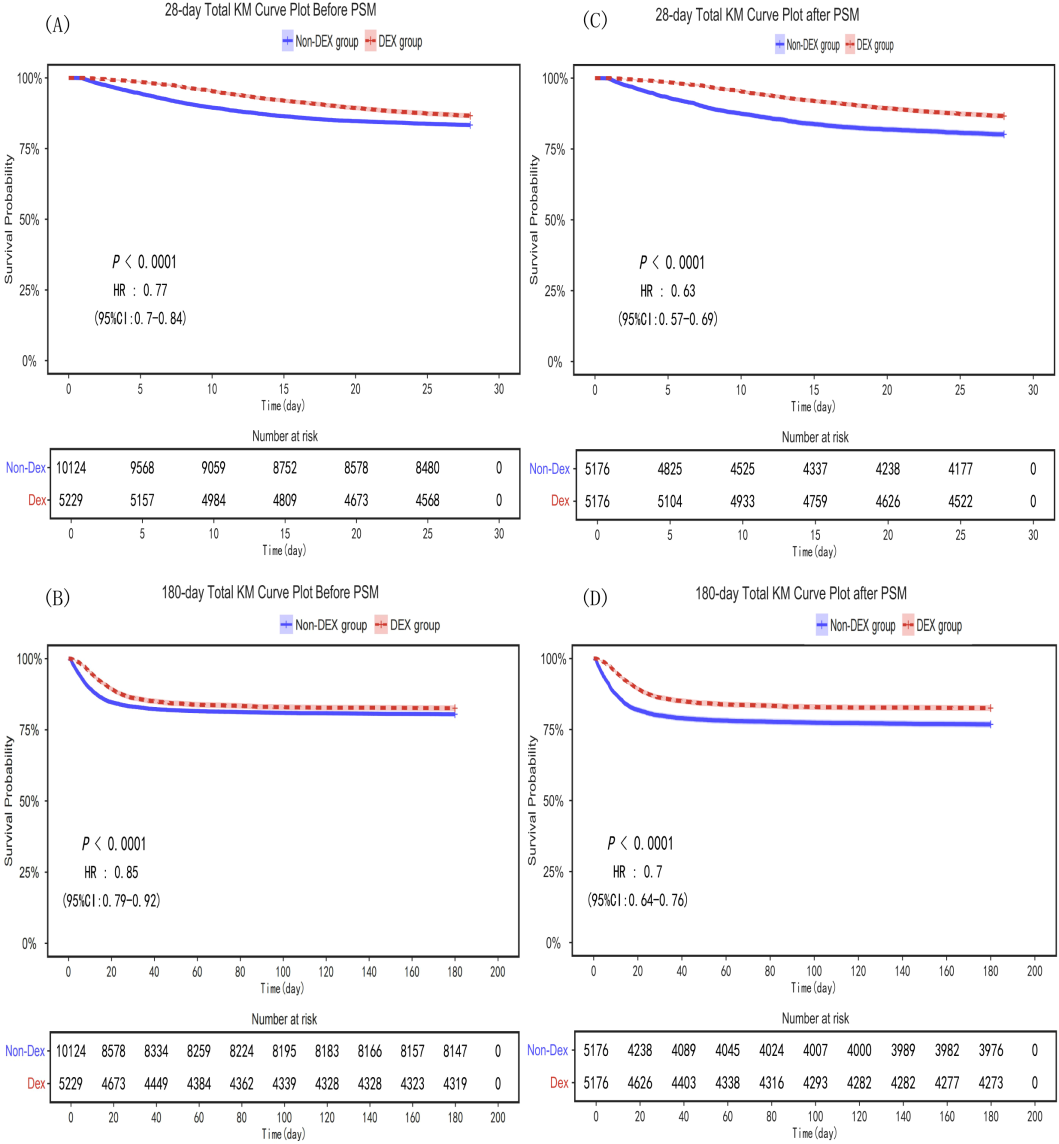
Kaplan‒Meier survival curves of the two groups for the 28-day and 180-day mortality risks before and after PSM. **(A)** 28-day Total KM Curve Plot Before PSM, **(B)** 28-day Total KM Curve Plot after PSM, **(C)** 180-day Total KM Curve Plot Before PSM, **(D)** 180-day Total KM Curve Plot Before PSM. PSM, propensity score matching; DEX, dexmedetomidine.

LASSO regression revealed no problems of covariance, as shown in **Supplementary Table S2**. Kaplan-Meier analysis showed lower 28-day all-cause mortality in the DEX group compared with the Non-DEX group (HR: 0.77, 95% CI: 0.70–0.84, *P* < 0.001) (**Figure 3A**). Cox analysis revealed that, compared with membership in the Non-DEX group, membership in the DEX group was associated with a reduced risk of 28-day all-cause mortality in the uncorrected model (13.39% vs. 16.66%, HR: 0.766, 95% CI: 0.701–0.837, *P* < 0.001), partially corrected model (HR: 0.752, 95% CI: 0.688–0.822, *P* < 0.001), and fully corrected model (HR: 0.615, 95% CI: 0.60–0.675, *P* < 0.001) (**Tables 2**, **3**). Furthermore, compared with the Non−DEX group, the DEX group showed lower 180−day all−cause mortality (HR: 0.85, 95% CI: 0.79–0.92, *P* < 0.001) (**Figure 3B**). Dexmedetomidine use reduced the 180-day risk of all-cause mortality according to the uncorrected model (17.40% vs. 19.53%; HR: 0.852, 95% CI: 0.788–0.922, *P* < 0.001), partially corrected model (HR: 0.838, 95% CI: 0.774–0.907, *P* < 0.001), and fully corrected model (HR: 0.652, 95% CI: 0.601–0.708, *P* < 0.001) (**Tables 2**, **3**).

**Table 2:** Survival results of the two groups before and after PSM.

Categories	28-day all-cause mortality	180-day all-cause mortality
Before PSM	HR (95% CI, *P* value)	HR (95% CI, *P* value)
Model l	0.766 (0.701-0.837, < 0.001)	0.852 (0.788-0.922, < 0.001)
Model 2	0.752 (0.688-0.822, < 0.001)	0.838 (0.774-0.907, < 0.001)
Model 3	0.615 (0.600-0.675, < 0.001)	0.652 (0.601-0.708, < 0.001)
After PSM	HR (95% CI, *P* value)	HR (95% CI, *P* value)
Model 1	0.629 (0.571-0.693, < 0.001)	0.701 (0.643-0.765, < 0.001)
Model 2	0.611 (0.555-0.673, < 0.001)	0.682 (0.626-0.744, < 0.001)
Model 3	0.595 (0.539-0.656, < 0.001)	0.632 (0.580-0.690, < 0.001)

HR, hazard ratio; CI, confidence interval; PSM, propensity score matching.

Model 1: uncorrected model.

Model 2: partially corrected model, adjusted only for age, ethnicity, and SOFA score.

Model 3: fully corrected model, adjusted for confounding variables with a coeff_lamda≠0 according to LASSO regression analysis.

**Table 3:** Outcome indicators of the two groups before and after PSM.

Categories	Before PSM				After PSM			
	DEX group (n = 5229)	Non-DEX group (n = 10124)	SMD	*P*	DEX group (n = 5176)	Non-DEX group (n = 5176)	SMD	*P*
28-day all-cause mortality, n (%)	700.00 (13.39)	1687.00 (16.66)	0.09	< 0.001	693.00 (13.39)	1027.00 (19.84)	0.17	< 0.001
180-day all-cause mortality, n (%)	910.00 (17.40)	1977.00 (19.53)	0.05	0.001	903.00 (17.45)	1200.00 (23.18)	0.14	< 0.001
Hospital LOS (days)	15.12 (8.67-25.99)	9.31 (5.77-16.35)	-0.44	< 0.001	15.08 (8.62-25.93)	10.20 (6.00-18.49)	-0.34	< 0.001
ICU LOS (days)	6.84 (3.23-13.15)	3.52 (1.95-7.07)	-0.48	< 0.001	6.81 (3.20-13.10)	4.00 (2.11-8.16)	-0.36	< 0.001
Mechanical ventilation duration (hours)	78.43 (34.67-160.00)	45.00 (22.08-91.88)	-0.39	< 0.001	78.00 (34.50-159.43)	51.00 (24.00-107.60)	-0.28	< 0.001
IFD_28 (days)	19.95 (7.64 - 24.53)	23.81 (14.53 - 25.91)	0.25	< 0.001	19.99 (7.75 - 24.56)	22.84 (7.14 - 25.71)	0.11	< 0.001
VFD_28 (days)	24.10 (17.67 - 26.40)	25.75 (21.17 - 27.00)	0.10	< 0.001	24.11 (17.71 - 26.40)	25.24 (17.34 - 26.92)	-0.02	< 0.001

PSM, propensity score matching; ICU, intensive care unit; SMD, standardized mean difference; LOS, length of stay; IFD_28, ICU-free days at 28 days; VFD_28, ventilator-free days at 28 days.

After PSM, as shown in the results of Kaplan–Meier analysis in **Figure 3C**, the 28-day mortality rate was significantly greater in the Non-DEX group than in the DEX group (HR: 0.63, 95% CI: 0.57–0.69, *P* < 0.001). Consistent with the pre-PSM results, membership in the dexmedetomidine group was associated with a reduced risk of 28-day mortality according to the uncorrected model (13.39% vs. 19.84%, HR: 0.629, 95% CI: 0.571–0.693, *P* < 0.001), partially corrected model: (HR: 0.611, 95% CI: 0.555–0.673, *P* < 0.001), and fully corrected model (HR: 0.595; 95% CI: 0.539–0.656, *P* < 0.001). The Kaplan–Meier plot in **Figure 3D** shows that the 180-day mortality rate was significantly greater in the Non-DEX group than in the DEX group (HR: 0.7, 95% CI: 0.64–0.76, *P* < 0.001). Dexmedetomidine use reduced the risk of 180-day all-cause mortality according to the uncorrected model (17.45% vs. 23.18%, HR: 0.701, 95% CI: 0.643–0.765, *P* < 0.001), partially corrected model (HR: 0.682, 95% CI: 0.626–0.744, *P* < 0.001), and fully corrected model: (HR: 0.632, 95% CI: 0.58–0.69, *P* < 0.001) (**Tables 2**, **3**).

### 3.3 Dexmedetomidine use prolongs hospital LOS, ICU LOS and duration of mechanical ventilation in patients with sepsis, Dexmedetomidine was associated with reductions in both 28-day ventilator-free days and ICU-free days.

Before PSM, dexmedetomidine use was associated with a longer hospital LOS (median 15.12 days vs. 9.31 days, *P* < 0.001) and a longer ICU LOS (median 6.84 days vs. 3.52 days, *P* < 0.001). In addition, the duration of mechanical ventilation was significantly longer in the DEX group than in the Non-DEX group (median 78.43 h vs. 45.00 h, *P* < 0.001). After PSM, dexmedetomidine use remained associated with a longer hospital LOS (median 15.08 days vs. 10.20 days, *P* < 0.001) and a longer ICU LOS (median 6.81 days vs. 4.00 days, *P* < 0.001). In addition, the duration of mechanical ventilation remained significantly longer in the DEX group than in the Non-DEX group (median 78.00 h vs. 51.00 h, *P* < 0.001) (**Table 3**).

To address potential bias arising from differences in survival and follow-up time when interpreting durations of hospitalization and mechanical ventilation, we analyzed the composite endpoints of ventilator-free days and ICU-free days at 28 days, which account for both mortality and resource use. In the composite endpoint analysis (incorporating mortality and recovery rate), the DEX group after PSM showed significantly shorter median ventilator-free days (median 24.11 days vs. 25.24 days, *P* < 0.001) and ICU-free days at 28 days (median 19.99 days vs. 22.84 days, *P* < 0.001) compared to controls (**Table 3**).

### 3.4 Exposure-response and infusion rate-response relationships between dexmedetomidine administration and survival outcomes

When comparing different durations of exposure, dexmedetomidine administered for >0 to ≤48 hours was associated with a reduction in 28-day and 180-day all-cause mortality. In contrast, prolonged use (>48 hours) showed no significant effect on these survival outcomes (**Table 4**; **Supplementary Table S3**).

**Table 4:** Dexmedetomidine administration: exposure-response and infusion rate-response relationships with outcomes after PSM.

Categories	n(%)	28-day all-cause mortality	180-day all-cause mortality
Duration (hours)		HR (95%CI, *P* value)	HR (95%CI, *P* value)
0	5246 (50.70)	1	1
24≥duration>0	2083 (20.10)	0.900(0.856-0.947, <0.001)	0.891(0.847-0.937, <0.001)
48≥duration>24	1138 (11.00)	0.923(0.866-0.985, 0.015)	0.917(0.860-0.978, 0.008)
>48	1885 (18.20)	0.963(0.913-1.015, 0.156)	0.990(0.939-1.044, 0.716)
Infusion Rate (μg/kg/h)		HR (95%CI, *P* value)	HR (95%CI, *P* value)
0	5176 (50.00)	1	1
0.3≥Rate>0	698 (6.70)	0.858(0.793-0.929, <0.001)	0.846(0.781-0.915, <0.001)
0.6≥Rate>0.3	1945 (18.80)	0.924(0.877-0.974, 0.003)	0.918(0.871-0.967, 0.001)
>0.6	2533 (24.50)	0.949(0.905-0.996, 0.032)	0.968(0.0.923-1.015, 0.180)

PSM, propensity score matching; HR, hazard ratio; CI, confidence interval.

When comparing different infusion rates, a dexmedetomidine rate of > 0 to ≤0.6 μg/kg/h was associated with lower 28-day and 180-day all-cause mortality compared with the non−dexmedetomidine group, whereas no significant survival benefit was observed at rates exceeding 0.6 μg/kg/h (**Table 4**; **Supplementary Table S3**).

### 3.5 Sensitivity analysis reveals the robustness of the Cox regression model

Among mechanically ventilated patients with sepsis and SOFA scores greater than 8, the risk of 28-day all-cause mortality was lower in the DEX group than in the Non-DEX group according to the uncorrected model (20.45% vs. 33.02%, HR: 0.85, 95% CI: 0.796–0.907, *P* < 0.001), partially corrected model (HR: 0.855, 95% CI: 0.801–0.914, *P* < 0.001), and fully corrected model (HR: 0.837, 95% CI: 0.781–0.897, *P* < 0.001). Dexmedetomidine use reduced 180-day all-cause mortality according to the uncorrected model (26.52% vs. 37.35%, HR: 0.857, 95% CI: 0.803–0.915, *P* < 0.001), the partially corrected model (HR: 0.864, 95% CI: 0.809–0.923, *P* < 0.001) and the fully corrected model (HR: 0.831, 95% CI: 0.775–0.89, *P* < 0.001) (**Supplementary Tables S4**, **S5**). Dexmedetomidine use was also associated with longer hospital LOS (median 18.21 days vs. 10.79 days, *P* < 0.001) and ICU LOS (median 8.88 days vs. 4.88 days, *P* < 0.001). In addition, the duration of mechanical ventilation was significantly longer in the dexmedetomidine group (median 106.00 h vs. 63.97 h, *P* < 0.001) (**Supplementary Table S5**).

After propensity score matching, the results remained consistent. specifically, the use of dexmedetomidine was associated with a reduced risk of 28-day mortality in the uncorrected model (20.45% vs. 36.54%, HR: 0.47, 95% CI: 0.407–0.542, *P* < 0.001), partially corrected model (HR: 0.479, 95% CI: 0.415–0.553, *P* < 0.001), and fully corrected model (HR: 0.506, 95% CI: 437–0.587, *P* < 0.001). Dexmedetomidine use was also associated with a reduced 180-day risk of all-cause mortality in the uncorrected model (26.59% vs. 40.95%, HR: 0.542, 95% CI: 0.476–0.616, *P* < 0.001), partially corrected model (HR: 0.551, 95% CI: 0.485–0.628, *P* < 0.001), and fully model-corrected model (HR: 0.542, 95% CI: 0.475–0.62, *P* < 0.001) (**Supplementary Tables S4, S5**). Dexmedetomidine use was associated with a longer hospital LOS (median 18.17 days vs. 11.05 days, *P* < 0.001) and ICU LOS (median 8.86 days vs. 5.19 days, *P* < 0.001). In addition, the duration of mechanical ventilation was significantly longer in the dexmedetomidine group (median 105.75 h vs. 69.27 h, *P* < 0.001) (**Supplementary Table S5**).

In the fully adjusted model after excluding missing continuous variables, dexmedetomidine use before PSM was associated with a reduced risk of 28-day mortality (HR: 0.633, 95% CI: 0.578–0.694, *P* < 0.001). A similar reduction in 180-day all-cause mortality was also observed (HR: 0.664, 95% CI: 0.612–0.721, *P* < 0.001) (**Supplementary Table S6**). After PSM, the results remained consistent, The DEX group showed a lower risk of 28−day all−cause mortality than the Non-DEX group (HR: 0.920, 95% CI: 0.885–0.957, *P* < 0.001). Likewise, dexmedetomidine use was also associated with reduced 180−day all−cause mortality (HR: 0.654, 95% CI: 0.599–0.713, *P* < 0.001) (**Supplementary Table S6**).

Grouping propofol, a commonly used first−line sedative, with etomidate and midazolam into a single “Non-DEX” group may introduce bias due to pharmacological heterogeneity. To reduce this heterogeneity, we performed analyses based on specific drug combinations. In models stratified by propofol co−administration, both before and after propensity score matching, the dexmedetomidine−exposed groups (whether with or without propofol) consistently exhibited significantly lower 28−day and 180−day all−cause mortality compared with the group exposed to neither dexmedetomidine nor propofol (all *P* < 0.05) (**Supplementary Table S7**).

Patients in all three stages of AKI had higher 28−day and 180−day all−cause mortality compared with those without AKI (**Supplementary Table S8**), early administration of dexmedetomidine may provide greater clinical benefit.

### 3.6 Dexmedetomidine is significantly protective in most subgroups

**Figure 4:**
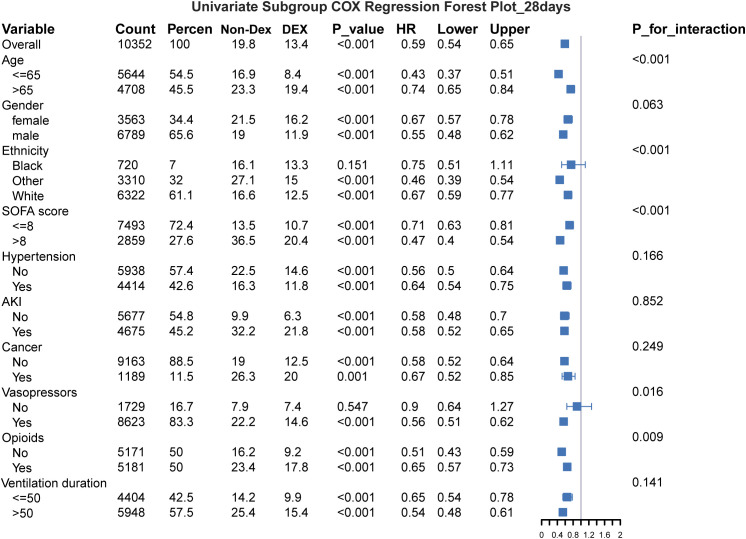
Forest plot of the relationships between dexmedetomidine and 28-day all-cause mortality. Adjusted for the use of antibiotics, use of glucocorticoids, systolic blood pressure, diastolic blood pressure, heart rate, respiration, oxygen saturation, arterial oxygen partial pressure, and arterial carbon dioxide partial pressure. SOFA, Sequential Organ Failure Assessment; AKI, acute kidney injury; DEX, dexmedetomidine; HR, hazard ratio; CI, confidence interval.

A total of 10352 patients on mechanical ventilation for sepsis were divided into separate subgroups on the basis of age (≤ 65 and > 65 years), sex, ethnicity, SOFA score (≤ 8 and > 8), hypertension, AKI, cancer, use of vasopressors, use of opioids, or duration of mechanical ventilation (≤ 50 h and > 50 h). Forest plots (**Figure 4**) depict the effect of dexmedetomidine on 28-day all-cause mortality in mechanically ventilated patients with sepsis for these subgroups. Our subgroup analyses revealed that dexmedetomidine had a significant protective effect for most of the different patient subgroups (*P* < 0.001).

In contrast, for patients who reported a black ethnicity (HR: 0.75, 95% CI: 0.51–1.11, *P* = 0.151) or who were not on vasopressor medications (HR: 0.90, 95% CI: 0.64–1.27, *P* = 0.547), dexmedetomidine did not show a significant protective effect.

### 3.7 Dexmedetomidine is an important factor associated with 28-day mortality in mechanically ventilated patients with sepsis

**Figure 5:**
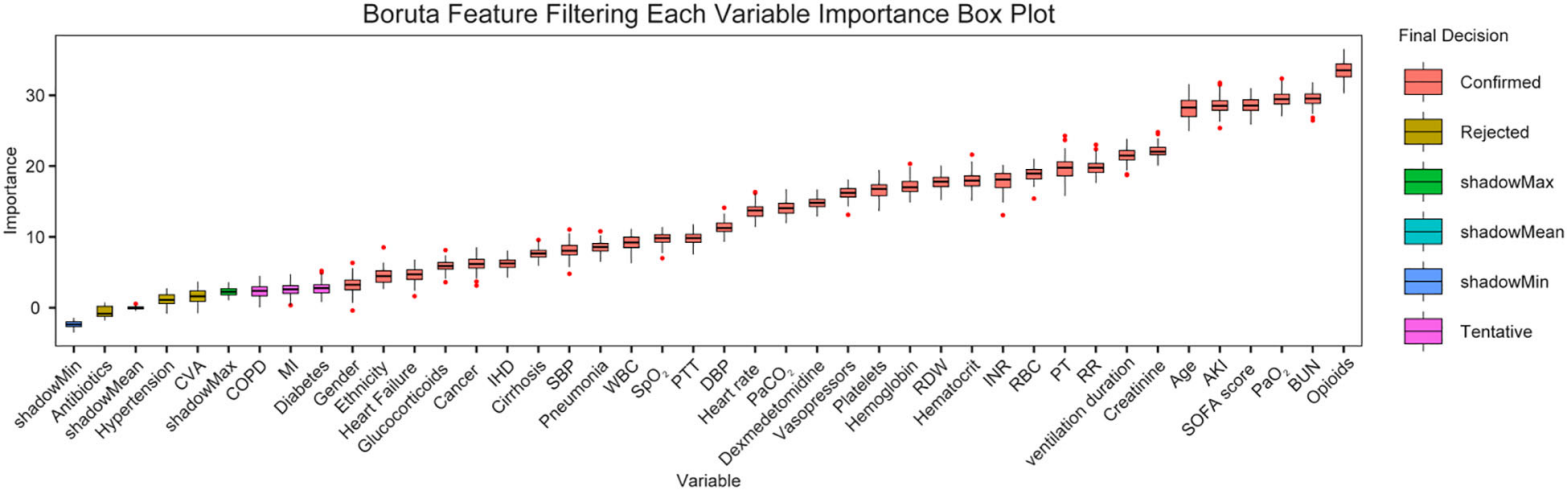
Feature selection based on the Boruta algorithm. The variables in the red boxes were identified as important features in the prediction of 28-day mortality. The horizontal axis shows the name of each variable, and the vertical axis represents the Z value of each variable. The box plot shows the Z values of each variable during model calculation. Red boxes represent important variables. BUN, blood urea nitrogen; PaO_2_, partial pressure of oxygen in arterial blood; SOFA, sequential organ failure assessment; AKI, acute kidney injury; ventilation duration, mechanical ventilation duration; RR, respiratory rate; PT, prothrombin time; RBC, red blood cell; INR, international normalized ratio; RDW, red blood cell distribution width; PaCO_2_, partial pressure of carbon dioxide in arterial blood; DBP, diastolic blood pressure; PTT, partial thromboplastin time; SpO_2_, saturation of peripheral oxygen; WBC, white blood cell; SBP, systolic blood pressure; IHD, ischemic heart disease; MI, myocardial infarction; COPD, chronic obstructive pulmonary disease; CVA, cerebrovascular accident.

Characteristic variables of the 15353 patients were subjected to the Boruta algorithm. **Figure 5** shows the results of the screening of important features associated with 28-day mortality in mechanically ventilated patients with sepsis. According to the results of the Boruta algorithm, in order of importance, the features that most contributed to the prediction of 28-day mortality were opioid use, BUN level, PaO_2_, SOFA score, AKI, age, creatinine level, mechanical ventilation duration, RR, PT, RBC count, INR, hematocrit, RDW, hemoglobin level, platelet count, vasopressor use, dexmedetomidine use, PaCO_2_, heart rate, DBP, PTT, SpO_2_, WBC count, pneumonia, SBP, cirrhosis, IHD, glucocorticoid use, heart failure, ethnicity, and sex.

### 3.8 A random forest model based on dexmedetomidine and other variables predicts outcomes in mechanically ventilated patients with sepsis

The data of the 15353 patients were divided into training and testing sets at a ratio of 7:3. The 20 most important features identified by the Boruta algorithm were used to build and validate five machine learning models, the parameter configurations of which are given in **Supplementary Table S9**. **Table 5** presents the detailed results of the five models, including metrics such as sensitivity, specificity, accuracy, positive predictive value (PPV), negative predictive value (NPV) and Brier score.

**Table 5:** Performance of each model in predicting 28-day mortality.

Model	AUC (95% CI)	Sensitivity	Specificity	Accuracy	PPV	NPV	Brier score
Training set (n = 10747)							
Random Forest	0.781(0.787-0.809)	0.751	0.695	0.704	0.313	0.938	0.208
Ctree	0.720(0.718-0.772)	0.605	0.761	0.736	0.320	0.912	0.234
GBM	0.673(0.683-0.710)	0.719	0.636	0.649	0.267	0.925	0.251
gamBoost	0.742(0.750-0.773)	0.676	0.718	0.711	0.307	0.923	0.218
Xgboost	0.723(0.706-0.730)	0.696	0.619	0.631	0.256	0.917	0.232
Testing set (n = 4606)							
Random Forest	0.811(0.814-0.844)	0.761	0.753	0.754	0.359	0.945	0.191
Ctree	0.719(0.725-0.760)	0.865	0.505	0.561	0.242	0.954	0.232
GBM	0.707(0.704-0.744)	0.769	0.597	0.623	0.258	0.934	0.243
gamBoost	0.748(0.749-0.785)	0.765	0.654	0.671	0.287	0.939	0.216
Xgboost	0.729(0.619-0.724)	0.379	0.881	0.677	0.688	0.674	0.228
eICU-CRD set (n = 3123)							
Random Forest	0.820(0.802-0.837)	0.659	0.824	0.783	0.556	0.878	0.181

AUC, area under the curve; 95% CI, confidence interval; PPV, positive predictive value; NPV, negative predictive value; Ctree, Conditional Inference Trees; GBM, Gradient Boosting Machines; gamBoost, Generalized Additive Model Boosting; Xgboost, eXtreme Gradient Boosting.

**Figure 6:**
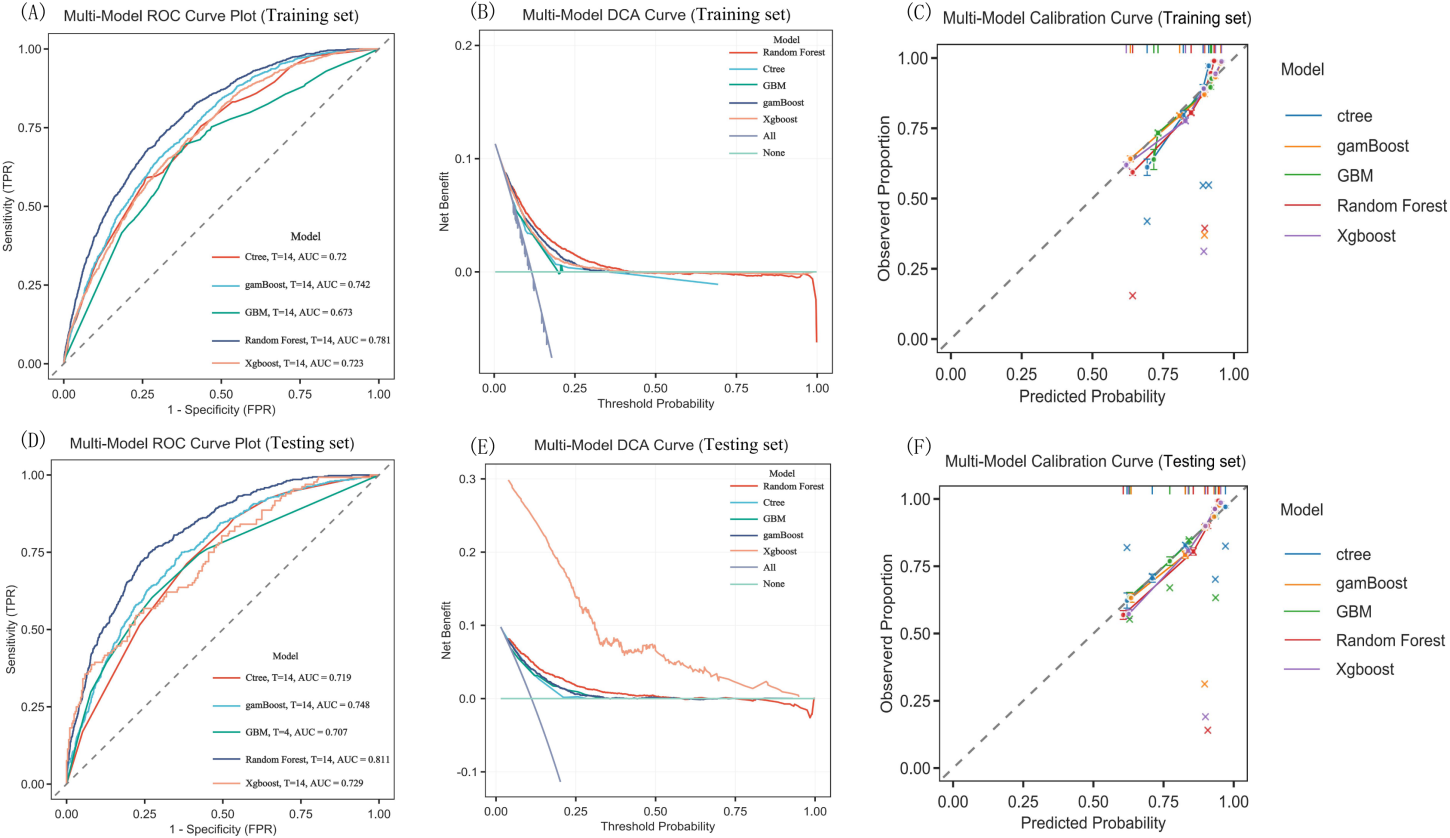
ROC curves, DCA plots and Calibration curve plots of the five machine learning models (MIMI-IV 3.0 database sets). **(A)** Multi-Model ROC Curve Plot of Training set, **(B)** Multi-Model ROC Curve Plot of Training set, **(C)** Multi-Model Calibration Curve of Training set, **(D)** Multi-Model ROC Curve Plot of Testing set, **(E)** Multi-Model ROC Curve Plot of Testing set, **(F)** Multi-Model Calibration Curve of Testing set. The calibration curve plot revealed good predictive accuracy bwteen the actual probability and predicted probability. T, time point; ROC, receiver operating characteristic; AUC, area under the curve; DCA, decision curve analysis; Ctree, Conditional Inference Trees; GBM, Gradient Boosting Machines; gamBoost, Generalized Additive Model Boosting; Xgboost, eXtreme Gradient Boosting.

In the training set, **Figure 6A** show the ROC curves of the five models, and model performance is quantified by the AUC values. Among them, the Random Forest model had the best performance, with an AUC of 0.781. According to the DCA plot **Figure 6B**, the machine learning models (especially the Random Forest model) significantly outperformed conventional strategies at threshold probabilities > 25%, demonstrating the clinical potential of these models in risk prediction. Despite not being the top-performing model in calibration analysis (**Figure 6C**), the Random Forest model nevertheless showed good predictive value. An AUC of 0.811 in the testing set further attests to the robust predictive performance of the Random Forest model. The Random Forest model attained an AUC of 0.811 in the testing set (**Figure 6D**). Accordingly, it demonstrates robust predictive performance (**Figures 6E, F**).

**Figure 7:**
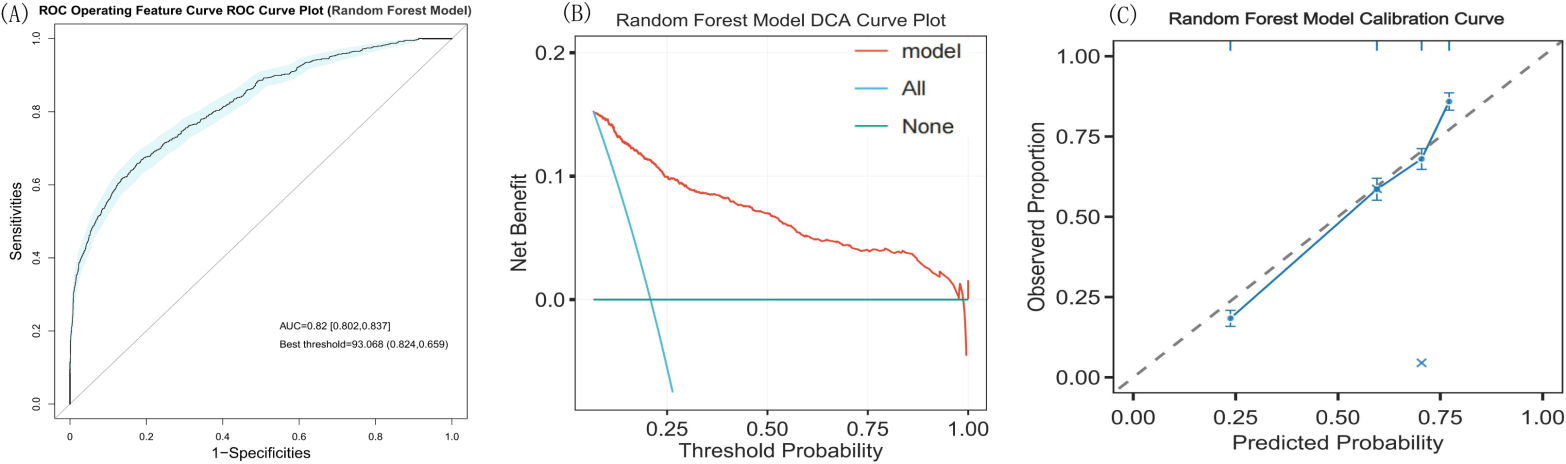
ROC curve, DCA plot and Calibration curve plot of the Random Forest model (eICU-CRD). **(A)** Random Forest Model ROC Curve Plot of eICU-CRD, **(B)** Random Forest Model DCA Curve Plot of eICU-CRD, **(C)** Random Forest Model Calibration Curve of eICU-CRD. The calibration curve plot revealed good predictive accuracy bwteen the actual probability and predicted probability. T, time point; ROC, receiver operating characteristic; AUC, area under the curve; DCA, decision curve analysis.

For external validation of the Random Forest model, 3123 patients from the eICU-CRD were included. Their baseline characteristics are presented in **Supplementary Table S10**. The model achieved an AUC of 0.820 (95% CI: 0.802–0.837) (**Table 5**), demonstrating good predictive performance (**Figure 7**).

### 3.9 AKI is an important factor affecting the outcomes of mechanically ventilated patients with sepsis after dexmedetomidine administration

**Figure 8:**
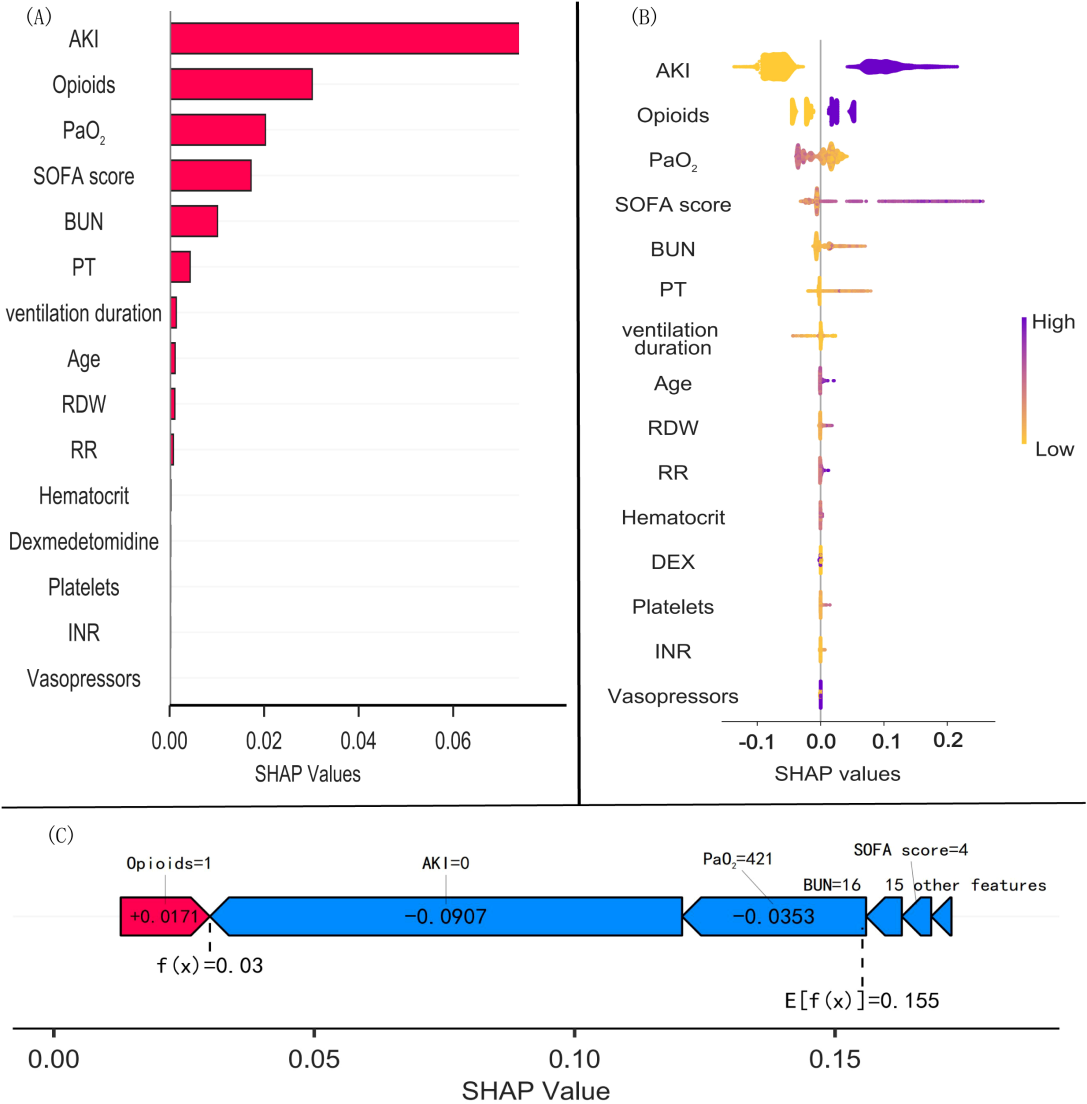
SHAP bar plot, beeswarm plot and force of variable importance (Random Forest model). **(A)** SHAP bar plot. **(B)** Beeswarm plot of Random Forest model: the horizontal position shows that the effect of the value is associated with a higher or lower prediction, and the colour showing the value is high (purple) or low (yellow) for that observation. **(C)** Force plot: red indicates elements that increase the risk of death, and blue indicates elements that decrease the risk of death. The length of the arrows indicates the degree of predicted impact: the longer the arrow is, the greater the impact. SHAP, SHapley Additive exPlanations; AKI, acute kidney injury; PaO_2_, partial pressure of oxygen in arterial blood; SOFA, sequential organ failure assessment; BUN: blood urea nitrogen; PT, prothrombin time; ventilation duration, mechanical ventilation duration; RDW, red blood cell distribution width; RR, respiratory rate; INR, international normalized ratio.

The SHAP algorithm was used to calculate the contributions of each feature variable to the predictions of the Random Forest model and to identify whether the associations between the predicted values and the target outcome were positive or negative. The variable importance plot (**Figure 8A**) depicts the factors in decreasing importance order: AKI had the strongest predictive value for all prediction levels, followed by opioid use, PaO_2_, and the SOFA score, while dexmedetomidine use ranked relatively poorly. **Figure 8B** reveals that the combination of AKI, the use of opioids, and a greater SOFA score has a positive effect, pushing the prediction towards mortality, whereas an increase in PaO_2_ has a negative effect, pushing the prediction towards survival. The force diagram of the SHAP values in **Figure 8C** indicates the prediction-related characteristics of individual patients and the contribution of each characteristic to the prediction of 28-day mortality. The use of opioids increases the risk of death, whereas uncomplicated AKI decreases the risk of death, with uncomplicated AKI having the greatest contribution to the prediction of mortality.

## 4 Discussion

### 4.1 Dexmedetomidine may reduce mortality in mechanically ventilated sepsis patients through multiple pathways

In this retrospective cohort study based on the MIMIC-IV (version 3.1) database, a total of 15353 mechanically ventilated patients with sepsis were enrolled. The 28-day all-cause mortality rate in the group receiving dexmedetomidine was 13.39%, whereas that in the group not receiving dexmedetomidine was 16.66%. After 1:1 PSM, the 28-day all-cause mortality rate was 13.39% in patients treated with dexmedetomidine, which remained significantly lower than that in patients who did not receive dexmedetomidine (19.84%), for a risk ratio of 0.595 (95% CI: 0.539–0.656, *P* < 0.001). Similarly, 180-day all-cause mortality was significantly lower in patients treated with dexmedetomidine (17.45% and 23.18% among nonusers of dexmedetomidine), for a risk ratio of 0.632 (95% CI: 0.58–0.69, *P* < 0.001). These results suggest that dexmedetomidine use significantly reduces short-term mortality in mechanically ventilated patients with sepsis.

The results of the present study are consistent with those of a large, retrospective study by Aso et al., who reported that dexmedetomidine use was associated with a reduction in 28-day mortality among 50671 mechanically ventilated patients with sepsis [OR: 0.78, 95% CI: 0.73–0.84; PSM: OR: 0.85, 95% CI: 0.80–0.91] (Aso et al., 2021). Several previous retrospective studies have also concluded that dexmedetomidine use reduces mortality in patients with sepsis (Zhang et al., 2019; Chen et al., 2020; Hu et al., 2022; Huang et al., 2024). However, a number of systematic meta-analyses have yielded results that are inconsistent with our findings, which showed that dexmedetomidine use did not reduce the 28-day mortality rate in mechanically ventilated patients with sepsis relative to controls (Huang et al., 2021; Wang et al., 2021; Ding et al., 2022; Liu et al., 2022). In addition, Zhang et al. found that, compared with benzodiazepine use but not isoproterenol use, dexmedetomidine use significantly reduced mortality among septic patients (Zhang et al., 2022). The reasons for these inconsistencies include several possibilities. First, some of the studies had small sample sizes, and some meta-analyses included studies of variable quality. Second, the range of interventions in the control group of each study was inconsistent, and the baseline characteristics of the population, the choice of sedation regimen and the setting of mechanical ventilation parameters were not always accounted for (Lee et al., 2021; Ge et al., 2023; Tao et al., 2023; Sheng et al., 2024; Wang et al., 2024; Zhang et al., 2025).

As a highly selective α_2_-adrenergic receptor agonist, dexmedetomidine exerts its protective effects through multiple, well-established mechanisms. For example, its anti-inflammatory actions include the modulation of inflammatory signaling pathways; the inhibition of NF-κB activation reduces the levels of proinflammatory cytokines (IL-6, TNF-α) (Zhang et al., 2018); while the blockade of the TLR-4/NF-κB pathway attenuates HMGB1-mediated neuroinflammation in the brain (Mei et al., 2021). Immune balance is preserved via the upregulation of Nur77 (Zhao et al., 2023) and the promotion of M2 macrophage polarization (Zhou et al., 2020), mitigating organ injury. Organ protection is achieved through stabilization of the cardiovascular system via suppression of sympathetic hyperactivity (Morelli et al., 2019), improvement in renal perfusion and filtration, and amelioration of AKI via KDM5A inhibition (Liu et al., 2019). In the central nervous system, dexmedetomidine maintains blood–brain barrier integrity (Yang et al., 2021) and reduces oxidative stress, improving sepsis-associated cognitive dysfunction. Dexmedetomidine-induced metabolic regulation further supports homeostasis by balancing glycolysis and oxidative stress pathways (Mei et al., 2021; Yang et al., 2021). These evidence-based mechanistic insights strongly corroborate the observed reductions in 28-day all-cause mortality in mechanically ventilated sepsis patients managed with dexmedetomidine, suggesting that the multimodal therapeutic effects of this drug underlie its survival benefits.

Our study revealed that dexmedetomidine significantly reduced 180-day all-cause mortality in mechanically ventilated patients with sepsis compared with controls (17.45% and 23.18%, HR: 0.632, 95% CI: 0.58–0.69, *P* < 0.001), whereas few relevant studies have reported the effect of dexmedetomidine on 180-day all-cause mortality. In a retrospective study by Wang et al. that included 15754 patients, dexmedetomidine use significantly reduced 180-day all-cause mortality in critically ill patients with AKI [22% vs. 27.2%, HR 0.77, 95% CI: 0.69–0.85], suggesting that the relevant mechanism of action may be related to the protective mechanism of dexmedetomidine on the kidneys (Wang et al., 2023). However, the effect of dexmedetomidine on 180-day mortality in septic patients needs to be verified with additional experimental studies.

### 4.2 Dexmedetomidine use and prolonged hospital stay and mechanical ventilation

Despite the superior performance of the dexmedetomidine group in terms of mortality, the median length of hospital (15.08 days) and ICU stay (6.81 days) were significantly longer than in the control group (10.2 days and 4.0 days). In addition, the duration of mechanical ventilation was significantly longer in the dexmedetomidine group than in the control group (median 78 h vs. 51.00 h, *P* < 0.001). This outcome pattern was further confirmed in an analysis of the composite endpoints of 28-day ventilator-free and ICU-free days, which account for mortality. In previous studies, the effects of dexmedetomidine use on hospital LOS, ICU LOS, and duration of mechanical ventilation were inconsistent.

According to Huang et al. (Huang et al., 2024), dexmedetomidine use was associated with a longer ICU stay (median 5.10 days vs. 6.22 days, *P* = 0.009), longer hospitalization (median 12.54 days vs. 14.87 days, *P* = 0.002), and a longer duration of mechanical ventilation (median 41.62 h vs. 48.00 h, *P* = 0.022), consistent with the results of Kai et al. (2016) and Hu et al. (2022) However, a meta-analysis by Liu et al. obtained very different results from ours, as they concluded that dexmedetomidine use shortened hospitalization time, ICU LOS, and duration of mechanical ventilation in mechanically ventilated patients with sepsis (Liu et al., 2022), aligning with the results of an RCT by Jakob (2012) and of a retrospective study by Aso et al. (2021). In addition, Zhang et al. (2019) reported that dexmedetomidine use did not differ from control treatments in terms of ICU LOS (WMD: 0.05; 95% CI, 0.590.48; *P* = 0.840) or duration of mechanical ventilation (WMD: 1.05; 95% CI, 0.272.37; *P* = 0.392).

We believe that the possible reasons for these seemingly contradictory results are that patients in the dexmedetomidine group were more likely to maintain a state of shallow sedation (i.e., higher Richmond Agitation-Sedation Scale scores), which may have delayed the process of withdrawal (Tripathi et al., 2017; Shehabi et al., 2019; Hughes et al., 2021). Second, studies suggest that dexmedetomidine may indirectly prolong the therapeutic window by reducing organ damage. In addition, differences in mechanical ventilation strategies (e.g., positive end-expiratory pressure levels, tidal volume settings) and the use of analgesic medications (e.g., fentanyl) were not standardized and may have had a confounding effect on the results. Additionally, the duration and dosage of dexmedetomidine administration may contribute to these findings.

Notably, despite prolonged mechanical ventilation, the reduced long-term mortality in the dexmedetomidine group suggests that it may have bought time for subsequent treatment by preserving organ function, which is closely related to the complexity of the pathophysiology of sepsis, and that attempting early withdrawal may overlook the potential risk of organ dysfunction.

### 4.3 Sensitivity analyses demonstrated the robustness of our findings

The sensitivity analysis conducted in this study revealed that among mechanically ventilated patients with sepsis with SOFA scores > 8 points, the results were consistent with the overall results in terms of 28-day all-cause mortality, 180-day all-cause mortality, length of hospital LOS, length of ICU LOS, and length of mechanical ventilation, thus validating the reliability of the results obtained in this study.

After excluding patients with missing data, the results remained consistent. Moreover, in an analysis comparing four medication subgroups, regardless of concomitant propofol use, dexmedetomidine was associated with lower 28−day and 180−day all−cause mortality compared with receiving neither dexmedetomidine nor propofol.

We found that the protective effect of dexmedetomidine was heterogeneous across different subgroups in this study. Specifically, dexmedetomidine use had a significant protective effect across most patient subgroups (*P* < 0.001), but patients of black ethnicity (HR: 0.75, 95% CI: 0.51–1.11, *P* = 0.151) as well as patients not on vasopressor medications (HR: 0.9, 95% CI: 0.64–1.27, *P* = 0.547) did not demonstrate a significant protective effect from the use of dexmedetomidine. In contrast, several studies have shown that dexmedetomidine reduces the need for vasopressor medication in patients with sepsis (Morelli et al., 2019; Cioccari et al., 2020; Li L. et al., 2023). The reasons for this may be related to inadequate sample sizes or differences in baseline characteristics. For example, Muszkat et al. noted that differences in the metabolism of α_2_ agonists in black patients may affect their efficacy (Muszkat et al., 2004). The sources of the observed heterogeneity may include sedative drug selection (propofol and midazolam use in the control group) and differences in mechanical ventilation strategies (e.g., uncontrolled positive end-expiratory pressure levels) (Zhou et al., 2025). These findings suggest that the clinical application of dexmedetomidine needs to be individualized with respect to the patient’s baseline characteristics and treatment strategy. Future studies should explore the dose-dependent effects of dexmedetomidine and precise application protocols.

### 4.4 Optimizing dexmedetomidine therapy: dose and duration dependence for mortality benefit

our findings indicate that the survival benefit of dexmedetomidine in mechanically ventilated patients with sepsis is dependent on both the duration of exposure and the rate of infusion. A finite treatment window (≤48 hours) and a moderated infusion rate (≤0.6 μg/kg/h) are associated with significant reductions in 28−day and 180−day all-cause mortality. Beyond these thresholds, no further survival advantage was observed. This suggests a potential “therapeutic window” for dexmedetomidine in this population, where exceeding optimal exposure parameters may not yield additional mortality benefit and could be associated with other clinical trade-offs, such as prolonged ventilation. Supporting this concept of a targeted therapeutic approach, sensitivity analyses stratified by AKI stage also affirmed the greater benefit of early dexmedetomidine administration.

Similarly, a study has shown that dexmedetomidine dosage and duration of administration are associated with reduced 28−day mortality in mechanically ventilated patients with sepsis (Huang et al., 2024). These results underscore the importance of precision in dosing and duration when utilizing dexmedetomidine for sepsis management in the ICU. Therefore, future studies should therefore aim to determine the optimal dosing regimen (including both dose and duration) of dexmedetomidine for mechanically ventilated patients with sepsis.

### 4.5 Dexmedetomidine administration may serve as a significant predictor of mortality in mechanically ventilated patients with sepsis

The result of feature selection by the Boruta algorithm in this study shows that, although dexmedetomidine was considered an important feature, the feature ranking was relatively low. This finding suggests that dexmedetomidine plays an important role in the outcomes of mechanically ventilated patients with sepsis, but this does not mean that it is a decisive factor. In this study, a comparison of the machine learning models revealed that the Random Forest model performed best, significantly outperforming traditional strategies at threshold probabilities of > 25%. The eICU-CRD dataset was used as an external validation cohort, confirming the reliability of the random forest model. Furthermore, in the analysis of the predictive values of the factors composing the Random Forest model using SHAP, we identified multiple important variables associated with 28-day all-cause mortality in mechanically ventilated patients with sepsis: AKI was found to be the most important predictor variable, followed by opioids, PaO_2_, and the SOFA score. Although dexmedetomidine was not among the most important predictors, dexmedetomidine is a sedative and not a therapeutic drug, and thus, when used as an adjunctive medication, does not play a decisive role in mechanically ventilated patients with sepsis; therefore, we consider the results of this study to be reasonable. Previous studies have similarly shown that AKI stage, oxygenation, and the SOFA score are key factors contributing to the predictive performance of machine learning algorithms for mortality in patients with sepsis (Fan et al., 2023; Gao et al., 2024). Li J. et al. (2023) reported that dexmedetomidine attenuated renal tubular iron death in sepsis-associated AKI by regulating the degradation of GPX4 via KEAP1, thereby exerting a renoprotective effect on the kidneys. Hu et al. reported that dexmedetomidine use reduced in-hospital mortality in critically ill patients with AKI in sepsis (Hu et al., 2022). Therefore, increased attention should be given to protecting renal function and avoiding nephrotoxic drugs in the sedation of mechanically ventilated patients with sepsis, perhaps via the use of dexmedetomidine over other options. In addition, the use of opioids was found to increase the risk of death in mechanically ventilated patients with sepsis; indeed, a recent basic experiment revealed that morphine exacerbates inflammation, behavior, and hippocampal structural deficits in septic rats (Ayieng’a et al., 2023), whereas opioid use may mask the pain-related stress response, which has been associated with an increased risk of infection (Abu et al., 2022); however, the precise role of use of opioids in mechanically ventilated patients with sepsis remains to be fully examined.

Although dexmedetomidine has shown positive results in mechanically ventilated patients with sepsis, its use requires caution. Some studies have reported bradycardia and hypotension as common adverse effects, which may limit its use in certain patients (Shehabi et al., 2019; Liu et al., 2022). Moreover, dexmedetomidine may increase the prevalence of arrhythmias, although its overall safety profile does not indicate that it significantly affects the occurrence of adverse events overall (Zhang et al., 2022). Furthermore, in a recent basic study involving septic rats treated with dexmedetomidine, Wang et al. reported that, instead of decreasing mortality in septic rats, compared with other sedatives (propofol, midazolam), dexmedetomidine treatment increased septic rat mortality by increasing in the levels of systemic and myocardial proinflammatory mediators, including TNF-α, IL-1β, IL-6, and VCAM-1 (Wang et al., 2025). Therefore, caution remains warranted in the choice of sedation regimen for mechanically ventilated patients with sepsis.

### 4.6 Study limitations and future research prospects

The main limitations of this study are as follows. Firstly, this research is inherently a retrospective observational study, with primary data sourced from the single-center MIMIC-IV database in the United States. Although we have enhanced the credibility of our models by performing external validation using the multi-center eICU-CRD database, differences in clinical practice, population characteristics, and resource distribution may still limit the direct generalizability of our findings to other healthcare systems or geographical regions. Secondly, retrospective data cannot fully eliminate residual confounding arising from unmeasured variations in clinical practice, such as sedation and analgesia strategies. Lastly, although we have strengthened the assessment of primary outcomes and reduced time-related biases by incorporating composite endpoints (28-day ventilator-free/ICU-free days) and competing risk analyses, the time-varying nature of both dexmedetomidine exposure and patient status remains an inherent methodological challenge in observational research.

Looking ahead, future research should focus on the following directions. First, within a prospective, multi-center framework, there is a need to systematically collect high-frequency, time-dependent covariate data and apply advanced causal inference methods, such as marginal structural models, to more accurately estimate the effects of dexmedetomidine. Second, well-designed randomized controlled trials are necessary to validate its impact on organ protection and long-term prognosis in broader populations. Finally, integrating multi-omics technologies with real-world data to elucidate the biological network of drug action will provide a solid foundation for achieving personalized sedation therapy in patients with sepsis.

## 5 Conclusions

Through large-scale real-world data analysis, this study confirmed that dexmedetomidine use significantly reduces short-term mortality in mechanically ventilated patients with sepsis, but its effects on prolonging hospitalization time, ICU stay and mechanical ventilation duration suggest that its short-term risks need to be weighed against its long-term benefits. To advance precision in sepsis care, further research should prioritize individualized dosing, dose optimization, and integrated multi-omics and time−to−event investigations.

## Data Availability

The datasets presented in this study can be found in online repositories. The names of the repository/repositories and accession number(s) can be found below: https://www.physionet.org/.
