# Supplementary material for: Exploring the association between dexmedetomidine and all-cause mortality in mechanically ventilated patients with sepsis through propensity score matching analysis and machine learning algorithms: a MIMIC-IV retrospective study
Source: Front Cell Infect Microbiol. 2026 Jan 26;15:1653883. doi: 10.3389/fcimb.2025.1653883 (PMC12883744; doi:10.3389/fcimb.2025.1653883)
Supplement: Supplementary file 1 [file DataSheet1.zip › Supplementary Material/Table S1.docx]

| Table S1 Missing number (%) for included variables in MIMIC-IV (v3.1) datasets. | |
| --- | --- |
| Variables | Missing Percent (%) |
| Age | 0.0 |
| SOFA score | 0.0 |
| WBC | 0.4 |
| RBC | 0.4 |
| Hematocrit | 0.5 |
| Hemoglobin | 0.4 |
| Platelets | 0.5 |
| RDW | 0.4 |
| Creatinine | 0.1 |
| BUN | 0.1 |
| INR | 2.7 |
| PT | 2.7 |
| PTT | 3.1 |
| PaCO_2_ | 3.1 |
| PaO_2_ | 3.0 |

Abbreviations: SOFA: sequential organ failure assessment; WBC: White Blood Cell Count, RBC: Red Blood Cell Count, RDW: Red Blood Cell Distribution Width, BUN: Blood Urea Nitrogen, INR: International Normalized Ratio, PT: prothrombin time, PTT: Partial Thromboplastin Time, PaCO_2_: Partial Pressure of Carbon Dioxide in Arterial Blood, PaO_2_: Partial Pressure of Oxygen in Arterial Blood.
