# Supplementary material for: Exploring the association between dexmedetomidine and all-cause mortality in mechanically ventilated patients with sepsis through propensity score matching analysis and machine learning algorithms: a MIMIC-IV retrospective study
Source: Front Cell Infect Microbiol. 2026 Jan 26;15:1653883. doi: 10.3389/fcimb.2025.1653883 (PMC12883744; doi:10.3389/fcimb.2025.1653883)
Supplement: Supplementary file 1 [file DataSheet1.zip › Supplementary Material/Table S10.docx]

| Table S10 Baseline characteristics of the two groups（eICU-CRD） | | | |
| --- | --- | --- | --- |
| Characteristics | DEX group  (n = 763) | Non-DEX group  (n = 2360) | *P* |
| Age (years) | 62.00 (51.00 - 72.00) | 65.00 (54.00 - 75.00) | < 0.001 |
| SOFA score | 9.00 (6.00 - 12.00) | 9.00 (6.00 - 11.00) | 0.248 |
| Laboratory tests |  |  |  |
| RBC count (m/µL) | 3.87 (3.44 - 4.29) | 3.74 (3.32 - 4.24) | 0.004 |
| Haematocrit (%) | 34.80 (31.20 - 38.80) | 34.15 (30.40 - 38.80) | 0.059 |
| Haemoglobin (g/dL) | 11.50 (10.10 - 12.80) | 11.20 (9.90 - 12.70) | 0.004 |
| Platelet count (K/µL) | 273.00 (183.00 - 372.00) | 244.00 (159.00 - 348.05) | < 0.001 |
| RDW (%) | 16.60 (15.00 - 18.90) | 16.70 (15.20 - 18.70) | 0.220 |
| Creatinine (mg/dL) | 1.76 (1.00 - 3.07) | 1.76 (1.03 - 3.18) | 0.761 |
| BUN (mg/dL) | 48.00 (28.00 - 71.00) | 42.00 (27.00 - 67.00) | 0.013 |
| INR (ratio) | 1.70 (1.30 - 2.51) | 1.70 (1.30 - 2.50) | 0.725 |
| PT (s) | 19.90 (15.90 - 26.20) | 19.00 (15.40 - 26.50) | 0.221 |
| PaCO_2_ (mmHg) | 49.40 (42.00 - 60.00) | 46.30 (39.00 - 55.00) | < 0.001 |
| PaO_2_ (mmHg) | 179.00 (130.00 - 265.00) | 161.00 (118.00 - 237.00) | < 0.001 |
| Vital signs |  |  |  |
| Heart rate (bpm) | 100.10 (88.00 - 116.00) | 100.00 (86.00 - 113.00) | 0.091 |
| Resp rate (bpm) | 23.00 (19.00 - 28.00) | 22.00 (18.00 - 25.90) | < 0.001 |
| Comorbidities, n (%) |  |  |  |
| AKI | 646.00 (84.67%) | 1,903.00 (80.64%) | 0.012 |
| Interventions, n (%) |  |  |  |
| Vasopressors | 536.00 (70.25%) | 1,656.00 (70.17%) | 0.967 |
| Opioids | 607.00 (79.55%) | 2,000.00 (84.75%) | < 0.001 |

Abbreviations: SOFA: Sequential Organ Failure Assessment; RBC: red blood cell; RDW: red blood cell distribution width; BUN: blood urea nitrogen; INR: international normalized ratio; PT: prothrombin time; PaCO_2_: partial pressure of carbon dioxide in arterial blood; PaO_2_: partial pressure of oxygen in arterial blood; Resp rate: respiratory rate; AKI: acute kidney injury.
