# Supplementary material for: Exploring the association between dexmedetomidine and all-cause mortality in mechanically ventilated patients with sepsis through propensity score matching analysis and machine learning algorithms: a MIMIC-IV retrospective study
Source: Front Cell Infect Microbiol. 2026 Jan 26;15:1653883. doi: 10.3389/fcimb.2025.1653883 (PMC12883744; doi:10.3389/fcimb.2025.1653883)
Supplement: Supplementary file 1 [file DataSheet1.zip › Supplementary Material/Table S2.docx]

| Table S2 Lasso survival status regression lambda value coefficient | | | | |
| --- | --- | --- | --- | --- |
| CoefName | Before PSM | | After PSM | |
|  | 28-days  coeff_lamda | 180-days  coeff_lamda | 28-days  coeff_lamda | 180-days  coeff_lamda |
| Gender | 0.0774 | 0.0683 | 0.1012 | 0.0899 |
| Age | 0.0213 | 0.0201 | 0.0231 | 0.0216 |
| Ethnicity | -0.1966 | -0.1596 | -0.2081 | -0.1713 |
| SOFA score | 0.0679 | 0.0495 | 0.0663 | 0.0454 |
| WBC | 0.0019 | 0.0018 | 0.0016 | 0.0020 |
| RBC | -0.2942 | -0.2893 | -0.2668 | -0.2646 |
| Hematocrit | 0.0597 | 0.0455 | 0.0506 | 0.0393 |
| Hemoglobin | -0.0183 | 0 | -0.0143 | -0.0005 |
| Platelets | 0.0006 | 0.0003 | 0.0005 | 0.0002 |
| RDW | 0.0703 | 0.0816 | 0.0680 | 0.0799 |
| Creatinine | -0.0111 | 0 | -0.0301 | -0.0148 |
| BUN | 0.0028 | 0.0026 | 0.0049 | 0.0041 |
| INR | 0.0105 | 0.0137 | 0.0630 | 0.0741 |
| PT | 0.0080 | 0.0073 | 0.0027 | 0.0016 |
| PTT | 0.0040 | 0.0036 | 0.0042 | 0.0034 |
| PaCO_2_ | -0.0011 | -0.0015 | 0.0022 | 0.0015 |
| PaO_2_ | -0.0030 | -0.0027 | -0.0025 | -0.0023 |
| SBP | 0.0003 | 0 | 0.0007 | 0.0003 |
| DBP | 0.0025 | 0.0017 | 0.0023 | 0.0015 |
| Heart rate | 0.0009 | 0.0013 | 0.0009 | 0.0013 |
| Resp rate | 0.0324 | 0.0299 | 0.0313 | 0.0290 |
| SpO_2_ | -0.0126 | -0.0099 | -0.0114 | -0.0075 |
| Hypertension | -0.0705 | -0.0866 | -0.0994 | -0.1141 |
| AKI | 0.5604 | 0.6094 | 0.5755 | 0.6499 |
| Cirrhosis | 0.2350 | 0.2671 | 0.2705 | 0.2992 |
| Pneumonia | 0.0985 | 0.1401 | 0.0973 | 0.1445 |
| CVA | 0.2828 | 0.2449 | 0.1512 | 0.1135 |
| Cancer | 0.1539 | 0.1460 | 0.1931 | 0.2089 |
| Diabetes | -0.1139 | -0.1004 | -0.1181 | -0.1126 |
| Heart Failure | -0.1135 | -0.0387 | -0.1341 | -0.0403 |
| MI | 0.3230 | 0.3015 | 0.3085 | 0.2647 |
| IHD | -0.1796 | -0.1695 | -0.2404 | -0.1977 |
| COPD | 0.1298 | 0.1164 | 0.1490 | 0.1187 |
| Antibiotics | 1.4790 | 1.4557 | 1.6409 | 1.4616 |
| Vasopressors | 0.5505 | 0.5860 | 0.5910 | 0.6791 |
| Glucocorticoids | 0.0984 | 0.1385 | 0.0980 | 0.1591 |
| opioids | 0.8065 | 0.8039 | 0.7622 | 0.7744 |
| mechanical ventilation  duration | -0.0013 | 0 | -0.0013 | 0 |

Abbreviations: PSM: propensity score matching; SMD: standardized mean difference; SOFA: sequential organ failure assessment; WBC: White Blood Cell Count, RBC: Red Blood Cell Count, RDW: Red Blood Cell Distribution Width, BUN: Blood Urea Nitrogen, INR: International Normalized Ratio, PT: prothrombin time, PTT: Partial Thromboplastin Time, PaCO_2_: Partial Pressure of Carbon Dioxide in Arterial Blood, PaO_2_: Partial Pressure of Oxygen in Arterial Blood; SBP: systolic blood pressure, DBP: diastolic blood pressure, Resp Rate: Respiratory Rate; SpO_2_: saturation of peripheral oxygen; AKI: Acute Kidney Injury, CVA: Cerebrovascular Accident, MI: Myocardial Infarction, IHD: Ischemic Heart Disease, COPD: Chronic Obstructive Pulmonary Disease.
