# Supplementary material for: Exploring the association between dexmedetomidine and all-cause mortality in mechanically ventilated patients with sepsis through propensity score matching analysis and machine learning algorithms: a MIMIC-IV retrospective study
Source: Front Cell Infect Microbiol. 2026 Jan 26;15:1653883. doi: 10.3389/fcimb.2025.1653883 (PMC12883744; doi:10.3389/fcimb.2025.1653883)
Supplement: Supplementary file 1 [file DataSheet1.zip › Supplementary Material/Table S3.docx]

| Table S3 Dexmedetomidine administration: exposure-response and infusion rate-response relationships with outcomes before PSM | | | |
| --- | --- | --- | --- |
| Categories | n（%） | 28-day all-cause mortality | 180-day all-cause mortality |
| duration(hours) |  | HR (95%CI, *P* value) | HR (95%CI, *P* value) |
| 0 | 10194 (66.40) | 1 | 1 |
| 24≥duration>0 | 2103 (13.70) | 0.593(0.515-0.682, <0.001) | 0.630(0.555-0.715, <0.001) |
| 48≥duration>24 | 1144 (7.50) | 0.740(0.625-0.876, <0.001) | 0.777(0.667-0.904, 0.001) |
| >48 | 1912 (12.50) | 0.978(0.868-1.101, 0.709) | 1.147(1.035-1.271, 0.009) |
| Infusion Rate (μg/kg/h) |  | HR (95%CI, *P* value) | HR (95%CI, *P* value) |
| 0 | 10124 (65.90) | 1 | 1 |
| 0.3≥Rate>0 | 700 (4.60) | 0.311(0.226-0.427, <0.001) | 0.370(0.283-0.348, <0.001) |
| 0.6≥Rate>0.3 | 1957 (12.70) | 0.749(0.657-0.854, <0.001) | 0.782(0.694-0.881, <0.001) |
| >0.6 | 2572 (16.80) | 0.907(0.814-1.011, 0.078) | 1.044(0.950-1.148, 0.372) |

Abbreviations: PSM: propensity score matching; HR: hazard ratio; CI: confidence interval.
