# Supplementary material for: Exploring the association between dexmedetomidine and all-cause mortality in mechanically ventilated patients with sepsis through propensity score matching analysis and machine learning algorithms: a MIMIC-IV retrospective study
Source: Front Cell Infect Microbiol. 2026 Jan 26;15:1653883. doi: 10.3389/fcimb.2025.1653883 (PMC12883744; doi:10.3389/fcimb.2025.1653883)
Supplement: Supplementary file 1 [file DataSheet1.zip › Supplementary Material/Table S4.docx]

| Table S4 Survival results of the two groups before and after PSM (patients with SOFA-score > 8) | | |
| --- | --- | --- |
| Categories | 28-day all-cause mortality | 180-day all-cause mortality |
| Before PSM | HR (95% CI, *P*-value) | HR (95% CI, *P*-value) |
| Model l | 0.85(0.796-0.907, < 0.001) | 0.857(0.803-0.915, < 0.001) |
| Model 2 | 0.855(0.801-0.914, < 0.001) | 0.864(0.809-0.923, < 0.001) |
| Model 3 | 0.837(0.781-0.897, < 0.001) | 0.831(0.775-0.89, < 0.001) |
| After PSM | HR (95% CI, *P*-value) | HR (95% CI, *P*-value) |
| Model 1 | 0.47(0.407-0.542, < 0.001) | 0.542(0.476-0.616, < 0.001) |
| Model 2 | 0.479(0.415-0.553, < 0.001) | 0.551(0.485-0.628, < 0.001) |
| Model 3 | 0.506(0.437-0.587, < 0.001) | 0.542(0.475-0.62, < 0.001) |

Abbreviations: PSM: propensity score matching; HR: hazard ratio; CI: confidence interval.

Model 1: uncorrected model.

Model 2: partially corrected model adjusted for Age; Ethnicity; SOFA score.

Model 3: fully corrected model adjusted for all variables selected in this study.
