# Supplementary material for: Exploring the association between dexmedetomidine and all-cause mortality in mechanically ventilated patients with sepsis through propensity score matching analysis and machine learning algorithms: a MIMIC-IV retrospective study
Source: Front Cell Infect Microbiol. 2026 Jan 26;15:1653883. doi: 10.3389/fcimb.2025.1653883 (PMC12883744; doi:10.3389/fcimb.2025.1653883)
Supplement: Supplementary file 1 [file DataSheet1.zip › Supplementary Material/Table S5.docx]

| Table S5 Outcome indicators of the two groups before and after PSM (patients with SOFA-score > 8) | | | | | | | | |
| --- | --- | --- | --- | --- | --- | --- | --- | --- |
| Categories | Before PSM | | | | After PSM | | | |
|  | DEX group  (n = 1452) | Non-DEX group  (n = 2423) | SMD | *P* | DEX group  (n = 1433) | Non-DEX group  (n = 1426) | SMD | *P* |
| 28-day all-cause mortality, n (%) | 297.00 (20.45) | 800.00 (33.02) | 0.29 | < 0.001 | 293.00 (20.45) | 521.00 (36.54) | 0.36 | < 0.001 |
| 180-day all-cause mortality, n (%) | 385.00 (26.52) | 905.00 (37.35) | 0.23 | < 0.001 | 381.00 (26.59) | 584.00 (40.95) | 0.31 | < 0.001 |
| Hospital LOS (days) | 18.21 (11.10 – 29.99) | 10.79 (6.13 – 19.22) | -0.46 | < 0.001 | 18.17 (11.08 – 29.90) | 11.05 (6.05 – 19.84) | -0.43 | < 0.001 |
| ICU LOS (days) | 8.88 (5.12 – 15.33) | 4.88 (2.65 – 8.99) | -0.51 | < 0.001 | 8.86 (5.08 – 15.24) | 5.19 (2.82 – 9.59) | -0.44 | < 0.001 |
| Mechanical ventilation  duration (hours) | 106.00 (53.00 – 201.34) | 63.97 (31.50 – 120.95) | -0.42 | < 0.001 | 105.75 (52.75 – 199.42) | 69.27 (33.20 – 138.00) | -0.34 | < 0.001 |

Abbreviations: PSM: propensity score matching; ICU: Intensive Care Unit; SMD: standardized mean difference; LOS: length of stay.
