# Supplementary material for: Exploring the association between dexmedetomidine and all-cause mortality in mechanically ventilated patients with sepsis through propensity score matching analysis and machine learning algorithms: a MIMIC-IV retrospective study
Source: Front Cell Infect Microbiol. 2026 Jan 26;15:1653883. doi: 10.3389/fcimb.2025.1653883 (PMC12883744; doi:10.3389/fcimb.2025.1653883)
Supplement: Supplementary file 1 [file DataSheet1.zip › Supplementary Material/Table S6.docx]

| Table S6 Survival results of the two groups before and after PSM (After removing missing variables) | | |
| --- | --- | --- |
| Categories | 28-day all-cause mortality | 180-day all-cause mortality |
| Before PSM | HR (95% CI, *P* value) | HR (95% CI, *P* value) |
| Model 3 | 0.633 (0.578-0.694, < 0.001) | 0.664 (0.612-0.721, < 0.001) |
| After PSM | HR (95% CI, *P* value) | HR (95% CI, *P* value) |
| Model 3 | 0.920 (0.885-0.957, < 0.001) | 0.654 (0.599-0.713, < 0.001) |

Abbreviations: PSM: propensity score matching; HR: hazard ratio; CI: confidence interval. Model 3: fully corrected model adjusted for all variables selected in this study.
