# Supplementary material for: Exploring the association between dexmedetomidine and all-cause mortality in mechanically ventilated patients with sepsis through propensity score matching analysis and machine learning algorithms: a MIMIC-IV retrospective study
Source: Front Cell Infect Microbiol. 2026 Jan 26;15:1653883. doi: 10.3389/fcimb.2025.1653883 (PMC12883744; doi:10.3389/fcimb.2025.1653883)
Supplement: Supplementary file 1 [file DataSheet1.zip › Supplementary Material/Table S7.docx]

| Table S7 Survival results of the four groups before and after PSM | | | |
| --- | --- | --- | --- |
| Categories | n（%） | 28-day all-cause mortality | 180-day all-cause mortality |
| Before PSM |  | HR (95%CI, *P* value) | HR (95%CI, *P* value) |
| Non-DEX+Non-P | 1390 (9.10) | 1 | 1 |
| DEX+P | 4854 (31.60) | 0.391(0.344-0.444, <0.001) | 0.460(0.409-0.517, <0.001) |
| DEX+Non-P | 650 (4.20) | 0.586(0.477-0.721, <0.001) | 0.622(0.514-0.752, <0.001) |
| Other combinations | 8459 (55.10) | 0.474(0.423-0.531, <0.001) | 0.501(0.450-0.557, <0.001) |
| After PSM |  | HR (95%CI, *P* value) | HR (95%CI, *P* value) |
| Non-DEX+Non-P | 708 (6.80) | 1 | 1 |
| DEX+P | 4805 (46.40) | 0.798(0.738-0.864, <0.001) | 0.795(0.735-0.861,0.001) |
| DEX+Non-P | 513 (5.00) | 0.872(0.778-0.976, 0.018) | 0.868(0.775-0.973, 0.015) |
| Other combinations | 4326 (41.80) | 0.850(0.785-0.920, <0.001) | 0.842(0.778-0.912, <0.001) |

Abbreviations: PSM: propensity score matching; HR: hazard ratio; CI: confidence interval;

DEX: dexmedetomidine; P: Propofol.
