# Supplementary material for: Exploring the association between dexmedetomidine and all-cause mortality in mechanically ventilated patients with sepsis through propensity score matching analysis and machine learning algorithms: a MIMIC-IV retrospective study
Source: Front Cell Infect Microbiol. 2026 Jan 26;15:1653883. doi: 10.3389/fcimb.2025.1653883 (PMC12883744; doi:10.3389/fcimb.2025.1653883)
Supplement: Supplementary file 1 [file DataSheet1.zip › Supplementary Material/Table S8.docx]

| Table S8 Subgroup analysis based on the AKI stage after PSM. | | | | |
| --- | --- | --- | --- | --- |
| Categories | DEX group  (n=5176) | Non-DEX group  (n=5176) | 28-day all-cause mortality | 180-day all-cause mortality |
| AKI stage | n（%） | n（%） | HR (95%CI, *P* value) | HR (95%CI, *P* value) |
| Without AKI | 2805.00  (54.19) | 2872.00  (55.49) | 1 | 1 |
| Ⅰ | 686.00  (13.25) | 707.00  (13.66) | 3.662(3.195-4.199, <0.001) | 3.795(3.349-4.300, <0.001) |
| Ⅱ | 1063.00  (20.54) | 918.00  (17.74) | 3.493(3.080-3.962, <0.001) | 3.855(3.441-4.319, <0.001) |
| Ⅲ | 622.00  (12.02) | 679.00  (13.12) | 3.943(3.440-4.520, <0.001) | 4.167(3.679-4.718, <0.001) |

Abbreviations: PSM: propensity score matching; HR: hazard ratio; CI: confidence interval; AKI: acute kidney injury.
