# Supplementary material for: Exploring the association between dexmedetomidine and all-cause mortality in mechanically ventilated patients with sepsis through propensity score matching analysis and machine learning algorithms: a MIMIC-IV retrospective study
Source: Front Cell Infect Microbiol. 2026 Jan 26;15:1653883. doi: 10.3389/fcimb.2025.1653883 (PMC12883744; doi:10.3389/fcimb.2025.1653883)
Supplement: Supplementary file 1 [file DataSheet1.zip › Supplementary Material/Table S9.docx]

| Table S9 Parameter configurations of five machine learning models | | | | | |
| --- | --- | --- | --- | --- | --- |
| Model | Random Forest | ctree | GBM | gamBoost | Xgboost |
| Parameter configurations | Minbucket: 3 | Minbucket: 7 | Shrinkage: 0.001 | Learning rate: 0.1 | Maxdepth: 6 |
|  | Maxdepth: 40 | Maxdepth: 30 | InteractionDepth: 1 | Base learner: bbs | Booster: gbtree |
|  | Mtry: 2 | Mtry: 2 | Nminobsinode: 10 | Degrees of freedom: 4 | Learning rate: 0.3 |
|  | Numtrees: 500 | MinCriterion: 0.95 | Numtrees: 100 |  | Minimum loss reduction: 0 |
|  | Minnodesize: 5 | Minsplit: 20 | BagFraction: 0.5 | Numtrees: 100 | L2 regularization coefficient: 1 |
|  | Ratio: 0.8 | Teststat: quadratic | Ratio: 0.8 | Ratio: 0.8 | Ratio: 0.8 |
|  | The sampling method applied to the training set is cross-validation (CV),  and the method used for the test set is holdout | | | | |

Abbreviations: Ctree: Conditional Inference Trees; GBM: Gradient Boosting Machines; gamBoost: Generalized Additive Model Boosting; Xgboost: eXtreme Gradient Boosting.
